# Using Polarized Spectroscopy to Investigate Order in Thin-Films of Ionic Self-Assembled Materials Based on Azo-Dyes

**DOI:** 10.3390/nano8020109

**Published:** 2018-02-15

**Authors:** Miguel R. Carro-Temboury, Martin Kühnel, Mariam Ahmad, Frederik Andersen, Ári Brend Bech, H. Krestian L. Bendixen, Patrick R. Nawrocki, Anders J. Bloch, Ilkay Bora, Tahreem A. Bukhari, Nicolai V. Bærentsen, Jens Carstensen, Smeeah Chima, Helene Colberg, Rasmus T. Dahm, Joshua A. Daniels, Nermin Dinckan, Mohamed El Idrissi, Ricci Erlandsen, Marc Førster, Yasmin Ghauri, Mikkel Gold, Andreas Hansen, Kenn Hansen, Mathias Helmsøe-Zinck, Mathias Henriksen, Sophus V. Hoffmann, Louise O. H. Hyllested, Casper Jensen, Amalie S. Kallenbach, Kirandip Kaur, Suheb R. Khan, Emil T. S. Kjær, Bjørn Kristiansen, Sylvester Langvad, Philip M. Lund, Chastine F. Munk, Theis Møller, Ola M. Z. Nehme, Mathilde Rove Nejrup, Louise Nexø, Simon Skødt Holm Nielsen, Nicolai Niemeier, Lasse V. Nikolajsen, Peter C. T. Nøhr, Dominik B. Orlowski, Marc Overgaard, Jacob Skaarup Ovesen, Lucas Paustian, Adam S. Pedersen, Mathias K. Petersen, Camilla M. Poulsen, Louis Praeger-Jahnsen, L. Sonia Qureshi, Nicolai Ree, Louise S. Schiermacher, Martin B. Simris, Gorm Smith, Heidi N. Smith, Alexander K. Sonne, Marko R. Zenulovic, Alma Winther Sørensen, Karina Sørensen, Emil Vogt, Andreas Væring, Jonas Westermann, Sevin B. Özcan, Thomas Just Sørensen

**Affiliations:** Nano-Science Center & Department of Chemistry, University of Copenhagen, Universitetsparken 5, 2100 København Ø, Denmark; martin.kuhnel@nano.ku.dk (M.K.); mah@science.ku.dk (M.A.); zgb268@alumni.ku.dk (F.A.); dfb556@alumni.ku.dk (Á.B.B.); skf209@alumni.ku.dk (H.K.L.B.); tbu@science.ku.dk (P.R.N.); gcf945@alumni.ku.dk (A.J.B.); ilkay.bora@gmail.com (I.B.); xrs483@alumni.ku.dk (T.A.B.); gtw317@alumni.ku.dk (N.V.B.); cgm648@alumni.ku.dk (J.C.); mnb838@alumni.ku.dk (S.C.); wkj412@alumni.ku.dk (H.C.); lvf878@alumni.ku.dk (R.T.D.); tqn207@ku.dk (J.A.D.); jdq431@alumni.ku.dk (N.D.); lnk170@alumni.ku.dk (M.E.I.); dgk483@alumni.ku.dk (R.E.); dhm812@alumni.ku.dk (M.F.); wqn556@alumni.ku.dk (Y.G.); fbm685@alumni.ku.dk (M.G.); wjg795@alumni.ku.dk (A.H.); rjf884@alumni.ku.dk (K.H.); bpg317@alumni.ku.dk (M.H.-Z.); tqn207@alumni.ku.dk (M.H.); vxk757@alumni.ku.dk (S.V.H.); rsz113@alumni.ku.dk (L.O.H.H.); pxv130@alumni.ku.dk (C.J.); smh645@alumni.ku.dk (A.S.K.); nhs827@alumni.ku.dk (K.K.); qwm886@alumni.ku.dk (S.R.K.); sgz864@alumni.ku.dk (E.T.S.K.); vtz390@alumni.ku.dk (B.K.); cts952@alumni.ku.dk (S.L.); sqv821@alumni.ku.dk (P.M.L.); chastine.munk@ind.ku.dk (C.F.M.); jzg995@alumni.ku.dk (T.M.); dtw667@alumni.ku.dk (O.M.Z.N.); bnw543@alumni.ku.dk (M.R.N.); gtf274@alumni.ku.dk (L.N.); djq495@alumni.ku.dk (S.S.H.N.); zvl379@alumni.ku.dk (N.N.); fwn674@alumni.ku.dk (L.V.N.); hgv633@alumni.ku.dk (P.C.T.N.); mnc612@alumni.ku.dk (D.B.O.); marc.overgaard@nano.ku.dk (M.O.); kvn491@alumni.ku.dk (J.S.O.); cfh645@alumni.ku.dk (L.P.); zwx958@alumni.ku.dk (A.S.P.); scd880@alumni.ku.dk (M.K.P.); bcr102@alumni.ku.dk (C.M.P.); bvh517@alumni.ku.dk (L.P.-J.); ptr511@alumni.ku.dk (L.S.Q.); cmh133@alumni.ku.dk (N.R.); nwr275@alumni.ku.dk (L.S.S.); dsk326@ku.dk (M.B.S.); dsk326@alumni.ku.dk (G.S.); cfh645@alumni.ku.dk (H.N.S.); zwx958@alumni.ku.dk (A.K.S.); markozenulovic@gmail.com (M.R.Z.); alma.soerensen@sund.ku.dk (A.W.S.); marc.overgaard@chem.ku.dk (K.S.); bvh517@ku.dk (E.V.); qwm886@ku.dk (A.V.); jdq431@ku.dk (J.W.); sgz864@ku.dk (S.B.Ö.)

**Keywords:** ionic self-assembly, thin films, molecular orientation, non-covalent forces, soft materials

## Abstract

Three series of ionic self-assembled materials based on anionic azo-dyes and cationic benzalkonium surfactants were synthesized and thin films were prepared by spin-casting. These thin films appear isotropic when investigated with polarized optical microscopy, although they are highly anisotropic. Here, three series of homologous materials were studied to rationalize this observation. Investigating thin films of ordered molecular materials relies to a large extent on advanced experimental methods and large research infrastructure. A statement that in particular is true for thin films with nanoscopic order, where X-ray reflectometry, X-ray and neutron scattering, electron microscopy and atom force microscopy (AFM) has to be used to elucidate film morphology and the underlying molecular structure. Here, the thin films were investigated using AFM, optical microscopy and polarized absorption spectroscopy. It was shown that by using numerical method for treating the polarized absorption spectroscopy data, the molecular structure can be elucidated. Further, it was shown that polarized optical spectroscopy is a general tool that allows determination of the molecular order in thin films. Finally, it was found that full control of thermal history and rigorous control of the ionic self-assembly conditions are required to reproducibly make these materials of high nanoscopic order. Similarly, the conditions for spin-casting are shown to be determining for the overall thin film morphology, while molecular order is maintained.

## 1. Introduction

To build a device from functional molecular materials, one has to rely on the materials to: (i) crystallize in a structure suitable for the fabrication of devices [1]; (ii) appropriately self-assemble or self-organize on the device substrate [2,3,4,5,6]; or you have to (iii) develop a tailor made processing platform such as the zone casting method that can order pentacene and hexabenzocoronene on substrates [7,8,9,10,11,12]. Shortcuts can be taken by combining (i) and (ii), as shown by the impressive accomplishments within the field of liquid crystals [13,14,15,16,17], while (ii) and (iii) can be combined in e.g., layer-by-layer assembly [18,19,20,21]. In this work, we use ionic self-assembly (ISA) [22,23,24,25,26,27], combining (i) and (ii) to be able to avoid (iii). 

ISA was first reported as an efficient method for layer-by-layer deposition of alternative charged species allowing thin films with lamellar order to be created by sequential dip coating [19,20,21,28,29]. The approach was later adopted to synthesize ordered nanomaterials directly from charged and hydrophobic molecular units upon precipitation [24,25,27,30,31,32,33,34,35,36]. Briefly, ISA happens when two solutions of a positively and a negatively charged water soluble building blocks are mixed. The individual building block is water soluble, but the complexes formed upon ion pairing of the building blocks are not. By choosing building blocks of the correct shape, lamellar structures can be precipitated directly following the ISA process [36].

**Figure 1:**
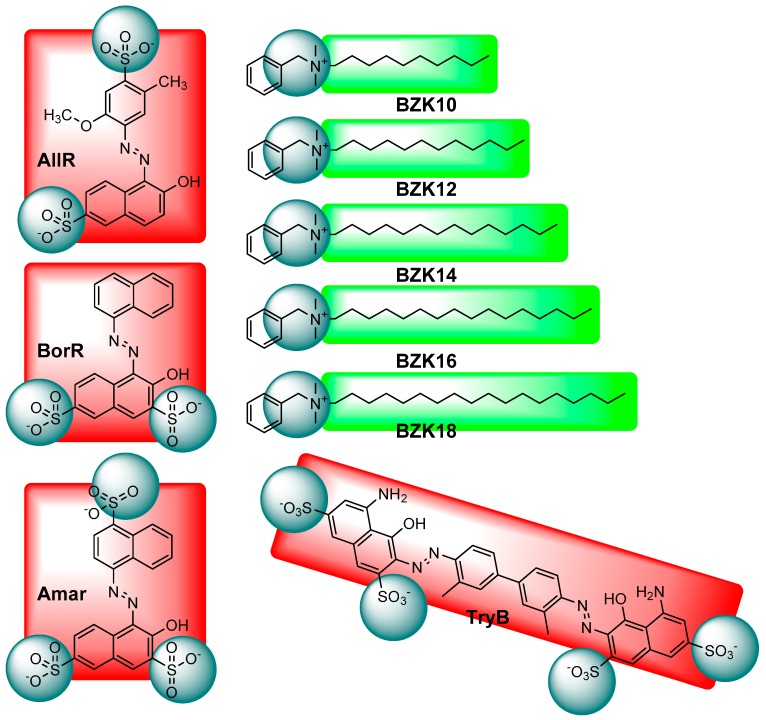
Functional units (red): Allura Red **AllR**, Bordeaux red **BorR**, Amaranth **Amar**, and Trypan Blue **TryB**; and benzalkonium surfactants **BZK** (green) used to make ionic self-assembled materials, the ionic groups are indicated in gray. All combinations of **BZK** and **AllR**, and **BorR**, and **Amar** were prepared, while only **TryB**:**BZK10** was prepared. Note that the surfactant exclusively varies in the length of the alkyl chain, from –(CH_2_)_9_CH_3_ **BZK10** to –(CH_2_)_17_CH_3_ **BZK18**.

Building on the seminal work of Faul and Antonietti [24,36], we have developed a ISA system based on benzalkonium surfactants **BZK** that allow for facile formation of ordered materials and thin films upon ISA with polyanionic functional units [37]. Here, we have undertaken a systematic investigation of three rectangular functional units (red) with five benzalkonium surfactant templates (green), only differentiated by an increasing chain length of the hydrophobic alkyl group from ten carbons in **BZK10** to eighteen in **BZK18** (see Figure 1). The three series of homolog materials were made from Allura Red **AllR**, Bordeaux Red **BorR**, and Amaranth **Amar** functional units. The results from these series were contrasted to a single ionic self-assembled material made from the rod shaped Trypan Blue **TryB** functional unit (see Figure 1).

**Figure 2:**
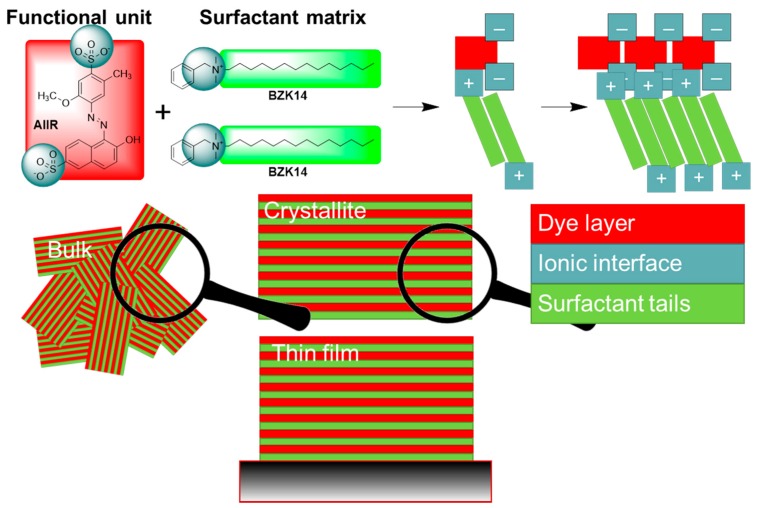
Cartoon representing the synthesis and the molecular structure of the homolog ionic self-assembled materials based on Allura Red, Bordeaux red, Amaranth and Trypan Blue anionic azo-dyes and cationic benzalkonium surfactants with alkane tail of varying length. The lamellar structure is randomly organized in bulk, but oriented in thin films. The lamellar may be divided in three sections, a dye layer (red), an ionic interface (gray), and spacing layer of alkyl chains (green). The systems studied here are identical, except the length of the alkyl chains of the surfactant used, which is assumed to give rise to a systematic increase in layer separation.

The materials were produced using ISA, as illustrated in Figure 2. As previously reported [37], the materials have a lamellar structure in bulk and thin films (see Figure 2). While investigations using X-ray diffraction (XRD) and atomic force microscopy (AFM) show that the materials in bulk and films have crystalline order, and that the material in thin films are ordered with respect to the substrate, these ordered thin films of the materials do not show activity in polarized optical microscopy [37]. A fact that implies: (a) the material is isotropic in the plane of the film [2,38]; or (b) the films are just a single domain. To challenge these assumptions, we decided to use polarized spectroscopy to probe the orientation of the dye molecules within the thin films.

Polarized spectroscopy was established to probe the orientation of transition moments [39] in the molecular scaffold, and to isolate the orientation of the individual molecules with respect to the laboratory coordinate system [40,41]. The methodology has been extended to probe dyes in ordered systems [42], and investigate methods of orienting molecules in polymer blends and the orientation of polymer backbones [2,43,44,45,46,47,48,49]. Here, we have updated the methodology of Michl and Thulstrup to be used with modern numerical methods [40,42]. Using polarized spectroscopy, we have determined the orientation of the transition moment with respect to the long axis of the molecule as defined by the angle ϕ_fz_ in the four dye molecules. Knowing the orientation of the transition dipole moment in the molecular framework, we can confirm the presence of order in the structure of the thin films using polarized absorption spectroscopy [37]. Thus, we were able to determine that the films made from all investigated materials have nanoscopic order, although we were not able to determine the exact molecular structure. While the results presented below do not allow for a clear answer to the question raised—whether the film is single domain or isotropic—we can conclude that polarized spectroscopy can be a valuable tool when investigating thin films with nanoscopic order. 

## 2. Results and Discussion

### 2.1. Synthesis

Ionic self-assembled (ISA) materials are often made by mixing a solution of a functional building block with a solution of a suitable surfactant, and then collecting the precipitating nanomaterial (see Figure 2) [22,23,25,37]. In the process of preparing materials from benzalkonium surfactants **BZK** with tail lengths from ten to eighteen, i.e. **BZK10**–**BZK18**, we observed that the precipitation kinetics varied. In some cases, gels rather than a fine precipitate were obtained. Therefore, a different approach was adopted, where the material was not collected as a precipitate, but was extracted using an organic solvent. We chose to use dichloromethane as the extracting solvent. The resulting solutions of nanomaterials were washed with water, dried over magnesium sulfate, and the pure products were isolated upon removal of the solvent. Despite rigorous drying, several materials were found to contain water in the structure (see Table 1). Of the sixteen materials prepared, only one was isolated in a form that was not clearly crystalline. While all other materials gave rise to information rich XRD data, this material (Table 1, entry 9) showed only a broad featureless background. 

A few of the entries correspond to previously reported materials [37]; for these, the amount of water can be compared between different methods of preparation. As the number of water molecules in the structure is different when the material is isolated by precipitation or extracted using an organic solvent, the structure must be able to accommodate a range of water molecules in the ordered structure. Table 1 shows which materials were isolated by precipitation and extraction, the water content does not vary according to whether material was extracted or precipitated. It is assumed that the water molecules are found in the ionic interface between the azo-dye building blocks and the surfactant head groups (see Figure 1). This will change the relative volumes of the constituent units of the structure, and will lead to changes in material properties. This is readily observed using XRD, although thermal annealing, which depends on time as well as temperature, effects may confuse the observations.

### 2.2. Bulk Structure

**Figure 3:**
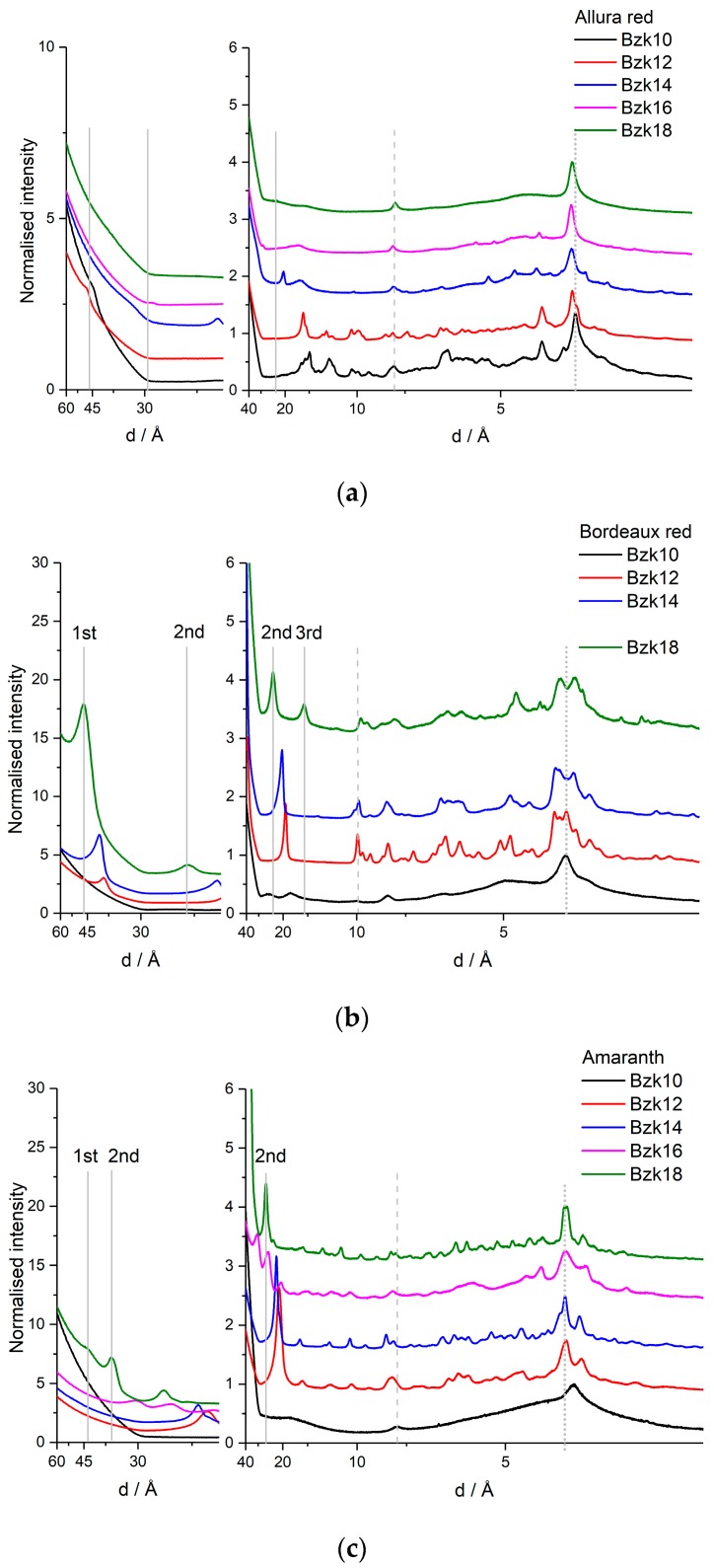
Powder XRD scattering curves for the three series of homolog ionic self-assembled materials based on: Allura Red (**a**); Bordeaux red (**b**); and Amaranth (**c**) anionic azo-dye functional units and benzalkonium surfactants with alkane tails of varying length from **BZK10** –(CH_2_)_9_CH_3_ to **BZK18** –(CH_2_)_17_CH_3_. The assumed principal axes are indicated with lines.

The lamellar structure of these materials was established previously [23,36,37]. Figure 3 shows the powder XRD scattering profiles for the three series of materials made from Allura Red, Bordeaux Red, and Amaranth. The materials are all crystalline. The lamellar repeat distance (gray line, Figure 3) is not observed for Allura Red (Figure 3a), while it is clearly evident in all materials made from Bordeaux Red (Figure 3b). For Amaranth (Figure 3c), a first order peak for the longest repeat distance cannot be distinguished, but a peak corresponding to a distance half that of the expected lamellar spacing is evident in the data for all five materials. This peak is interpreted as a second order Bragg peak arising from the lamella spacing. 

Note that there is only one peak in each series that change as the chain length of the surfactant is varied. The general scattering pattern is similar for all materials within a series, for Allura Red a higher degree of crystallinity is observed for materials made from surfactants with short tail lengths. For Bordeaux Red and Amaranth this trend is reversed, so that the shortest surfactant only shows few peaks in the scattering curve. The identified lamellar spacing is included in Table 2. 

As all materials are made from molecules of identical thickness, they all show a peak corresponding to a stacking distance of 0.4 nm (dotted gray line, Figure 3), assigned to a tilted stacking of the functional units. In this range, a hexagonal packing of the surfactant tails would also give rise to a peak. 

A constant within each series made from the same azo-dye is the width of the dye. Therefore, a peak related to the end-on-end packing of the dyes at a distance identical to or longer than the width of the dye molecules should be present in the data and independent of surfactant tail length. More than one peak may be found, but the one corresponding to the longest distance may be assigned to the width of the unit cell in the material (dashed gray line, Figure 3). Thus, the three principal axes may be assigned a priori, and a model for the molecular packing of the materials may be suggested that conform to these dimensions [37]. For **AllR** and **Amar** the dashed lines coincide with the width of the dye molecule on the shortest dimension. The size of the molecule can be determined from geometric considerations and molecular models. The models assume that the conjugated π-system of the molecules can be considered planar. Following this assumption, the molecules can be considered rectangular and thus described by two dimensions. That is, the dashed line corresponds to the shorter dimension for **AllR** and **Amar** and to the longer of the two dimensions for **BorR**.

The lamellar spacing is assumed to increase by a maximum of 0.3 nm per ethylene group for a material with fully extended alkyl chains at an angle perpendicular to the lamellar [5]. This is the distance observed for the one difference that can be determined for Allura Red (see Table 2). The distances increase of 0.2 nm consistently determined for Bordeaux Red suggest a more tilted geometry. There is no trend in the lamellar spacing determined for Amaranth. Small angle scattering, variable temperature experiments, and single crystal structures will be natural next step to take in the scrutiny of the solid-state structure of these materials. Note that all materials constitute a 3D lattice as well as two 2D lattices (see Figure 2).

### 2.3. Polarized Spectroscopy

Polarized light is an excellent probe for investigating ordered systems [40,41,42], as is well known from the use of polarized optical microscopy in the study of liquid crystalline systems [16]. Polarized microscopy requires anisotropy of the optical properties of the sample in the light path for the birefringence to occur, in the materials studied here the lamellar are either single crystalline or isotropic in the plane of the film. Either fact renders polarized microscopy useless [2]. The material is ordered in lamella, where the transition moment of the azo-dyes is highly ordered with respect to the surface normal of the substrate. Thus, polarized spectroscopy performed by tilting the substrate will give information of the orientation of the lamella, and the overall orientation of the dyes in the thin films.

The orientation of the optical transition moments in azo-dyes are far from trivial and more than one transition may be present in the main absorption band, note the β-naphtol derived dyes do not have *cis*-*trans* isomerism to consider [50]. To determine the orientation of the transition moments corresponding to the absorption band(s) of the four dyes used as building blocks, polarized spectroscopy was performed on the dyes in stretched polyvinyl alcohol films.

The strategy is the following: The angle between the long axis of the molecule, z, and its transition moment, **M_f_**, is noted ϕ_fz_, and is determined using the stretched polymers. Then, the angle between the transition moment **M_f_** and the surface normal of the thin films, Z, noted α_fZ_, is determined using the tilted plate method. With knowledge of these two angles, the two possible angles between the long axes of the azo-dyes and the surface normal of the thin film, ω_zZ_ and ω_zZ’_, are deduced. This angle is used to infer structural properties of the lamella. See Appendix A for a full walkthrough of the methodology. 

Note that if the value of α_fZ_ corresponds to magic angle (54.7°), it is not possible to distinguish if the transition moments are randomly oriented or aligned on average at 54.7°. Therefore, we can use the opposite argument to say that, if the angle is different from 54.7°, there exists a degree of alignment of the transition moments in the sample with respect to the surface normal of the thin films [43].

### 2.4. Stretched Polymers

**Figure 4:**
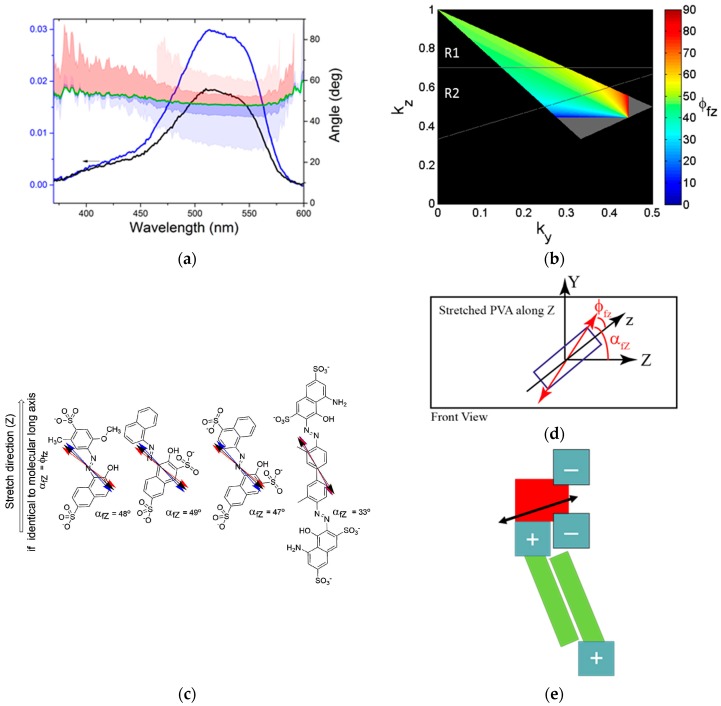
(**a**) Polarized absorption spectra of the sodium salt of Alura Red in stretched polyvinyl alcohol used for the determining α_fZ_ (note Z refers to the stretch direction) and ϕ_fz_, the angle ranges for ϕ_fz_ from the numerical treatment of the data are plotted in shades blue (assuming rod-shaped molecules) or red (assuming flat molecules); (**b**) the determined ϕ_fz_ from the polarized spectroscopy plotted in Thulstrup and Michl’s orientation triangle (for details, see the Supplementary Materials); (**c**) experimentally determined orientation of the transition moment **M_f_** respect to the stretching direction Z and the molecular long axis **z,** blue (assuming rod-shaped molecules), red (assuming flat molecules), and black (average); (**d**) sketch of the setup to measure stretched polymers; and (**e**) cartoon representation of the orientation of **M_f_** in the nanomaterial with dye (red), an ionic interface (gray), and alkyl chains (green).

To recap the seminal work of Thulstrup and Michl [41], the direction of the transition dipole moment **M_f_** may be related to the stretch direction of the polymer Z (see Figure 4). Note that, only in this section, the Z refers to the stretch direction of the polymer and not to the normal of the surface of the thin film. The data will allow the angle α_fZ_ between Z and **M_f_** to be determined (see Appendix A). A priori knowledge on the shape of the molecule enables the angle ϕ_fz_ between the transition moment **M_f_** and the long axis of the molecule **z** to be determined. The alternative is to investigate multiple transitions in each dye. Here, we use numerical methods to determine ϕ_fz_ from data on a single transition. The ϕ_fz_ angle can then be used to directly relate data obtained from polarized optical spectroscopy on thin films to the molecular structure, see below.

Figure 4a shows the data and the result from the modelling of the experimental data for Allura Red [40,41]. The α_fZ_ angle is well defined at 48°, while the ϕ_fz_ angle can vary from ≈30° to ≈60° at the emission maximum, depending on the assumptions made regarding the overall shape of the dye molecule. The assumptions regarding the molecular shape correspond to assuming that the molecules at perfect alignment can be located at a specific point of Thulstrup and Michl’s orientation triangle [40,41]. The relation between ϕ_fz_, the assumed shape of the molecule, and the orientation factor is plotted in Figure 4b, using Equation (A5). If the molecule is considered to be rod-like, it will follow the top line of the orientation triangle, while a flat-like molecule will be at the bottom edge of the orientation triangle. The numerical analysis allows us to determine the possible values ϕ_fz_ can take as a function of the position in the orientation triangle, as well as the most probable orientation of the transition dipole moment with regards to the long axis of the molecule, see the SI for detail. The result of the analysis is shown in Figure 4c. In short, the ϕ_fz_ range describes the possible orientations of the transition dipole moment of the primary transition in the three functional units. The variation is small, and we can use the fact that we know the ϕ_fz_ of the functional units to probe the thin films structure. As the spectra are only marginally perturbed between solution and thin films [37], we assume that the electronic transitions are similar in solution and in thin films and thus we can use ϕ_fz_ directly to determine the molecular orientation in the thin films following the cartoon representation in Figure 4e. 

### 2.5. Thin Film Structure

**Figure 5:**
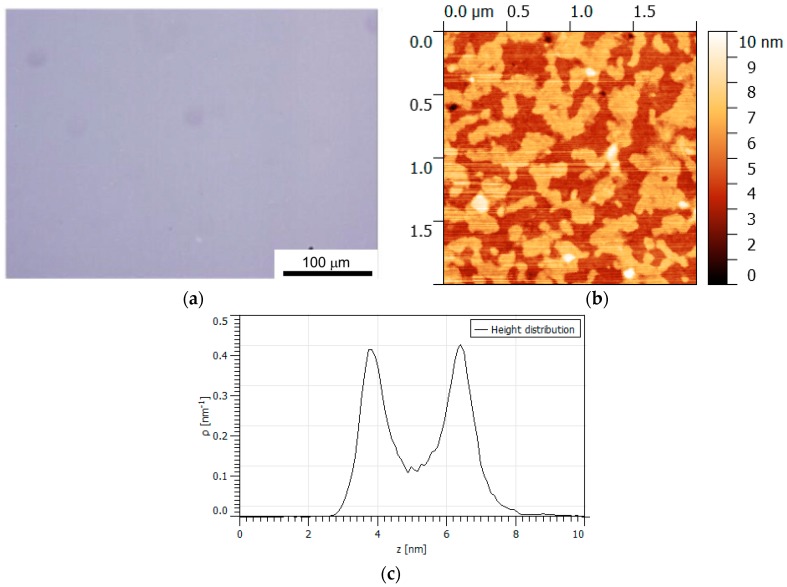
Microscopy data from a thin film made from a **AllR**:**BZK12** nanomaterial: (**a**) widefield optical microscopy image using 60× magnification; (**b**) atomic force micrograph showing the height differences in a 2 by 2 μm area of the film; and (**c**) height distribution corresponding to the entire image shown in (**b**).

The bulk materials were processed into thin films by dissolving the bulk material in dichloromethane and spin-casting onto a rotating glass substrate [2,37,38]. The morphology of the resulting films was homogeneous when observed in the optical microscope using widefield, darkfield and polarized illumination (see Figure 5 and the Supplementary Materials). The macroscopic film structure is homogeneous over the glass slide, without crystalline features or film defects. 

Investigating the nanoscopic order using AFM (see Figure 5 and the Supplementary Materials), the film morphology is shown to be dominated by a lamellar structure characterized by a data similar to those presented in Figure 5b with two or three layers clearly resolved (Figures S5, S6, S20, S21, S26, S27, S38, S39, S43, S44, S49 and S59). Some of the lamellar materials appear as uniform with holes in the form of layer defects and partially formed layers (Figures S30, S31, S34, S35, S62, S66, S69 and S70), while a few thin films are apparently amorphous partially formed films (Figures S49–S52, S55, S56, S75 and S76). Some films, particularly to Allura Red, appear to form with microcrystallites in or on top of the film (Figures S10–S13, S16 and S17). The crystallites cannot be observed in the optical microscope, but the crystallites give rise to films with a varied morphology on the nanoscale. These crystallites are most likely a result of the conditions used for spin-casting. The overall conclusion based on the AFM data is that a lamellar structure is the most prevalent structure exhibited in the thin film surface.

The height of apparent layers in the thin film was measured where possible. The results are included in Table 2, and show that the surface structure of the films correlates poorly with the distances found in the bulk structure. This is in stark contrast to what we have found previously [37], and leads us to find an alternative method to analyze the over-all molecular structure of the thin films. The crystalline materials could with great advantage be investigated using X-ray reflectometry [2,10,38,51]. Unfortunately, we found that useful X-ray reflectometry data could only be acquired using synchrotron radiation for the organic materials investigated here. Instead, we turned to polarized optical spectroscopy that, similar to X-ray reflectometry, probes the molecular order in the thin films.

### 2.6. Molecular Structure in Thin Films

**Figure 6:**
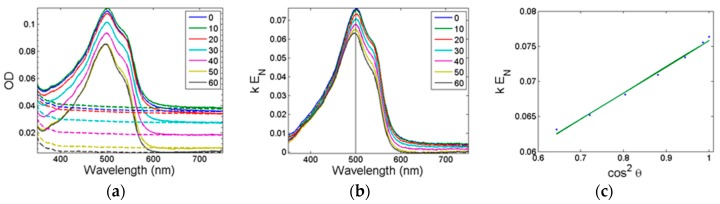
Data from polarized optical spectroscopy for Alura Red:**BZK18** thin film. (**a**) Optical density (raw data) of the sample with nanostructures (full line) and the glass blank sample (dashed line). The legend shows the incident angle θ_1_ from 0° to 60° in steps of 10°. (**b**) Absorption coefficient of the nanostructure E_N_ times the constant k. Only the central wavelength marked with the straight vertical line is used for calculating α_fZ,max_. (**c**) Linear regression (red line) of experimentally determined *kE_N_* (black dots) against cos^2^θ_1_ for the central wavelength.

**Figure 7:**
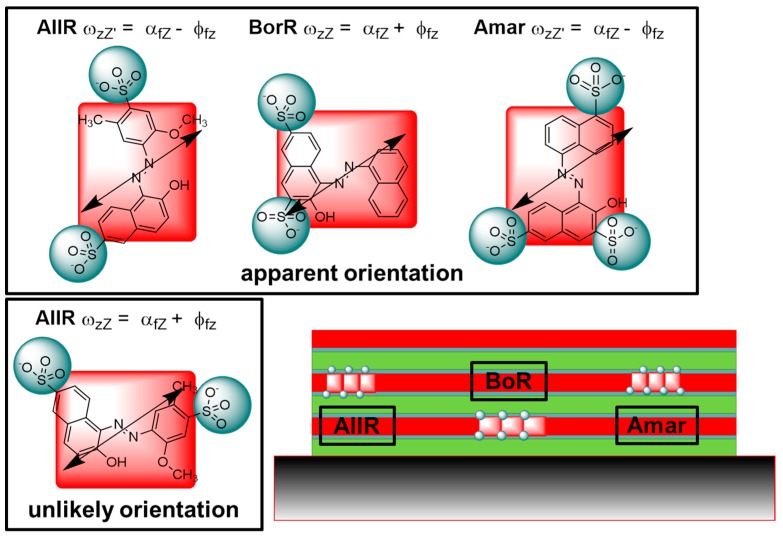
Molecular structure in thin films of the ionic self-assembled materials as determined by polarized absorption spectroscopy illustrated on a model of the lamellar structure with dye layers (red), ionic interface layers (gray), and layers of alkyl chains (green). The method is not able to differentiate between the two orientations shown for Allura Red **AllR**, but considerations on the lamella structure suggest that the orientation which positions the two charged groups at either side of the molecule would be preferred. The preferred orientation of Bordeaux Red **BorR** and Amaranth **Amar** is shown.

By measuring the absorption spectrum of the thin films as a function of the tilt angle θ using polarized light we can determine the average angle α_fZ_ between the substrate normal of the thin film, **Z**, and the transition dipole moment **M_f_** of the functional units. This was done for each material (see Figure 6 and Appendix A for the detailed theoretical treatment). The premise for the treatment is that the dye molecules are uniaxially ordered. For an isotropic material the corrected data would be identical for all tilt angles θ. For a perfectly aligned anisotropic material, the variation as a function of tilt angle θ will follow a simple cos^2^θ function [2]. Here, the variation is complicated and merit the full theoretical treatment described in Appendix A. The results are summarized in Table 2 and visualized in Figure 7. The analysis was for each dye performed at *λ*_max_ in solution and the resulting α_fZ,max_ was tabulated. For Allura Red and Bordeaux Red α_fZ,max_ varies from 55° to 63°, the variation is small yet systematic, indicating that the dye layer is influenced by the cohesive forces of the surfactant matrix. In contrast, α_fZ,max_ for Amaranth was determined at 64° ± 1°, suggesting that the tricationic dye dominates the structure in the material. This is consistent with the unsystematic variation of lamellar spacing determined in the bulk material. α_fZ,max_ is a measure of the average orientation of dye molecules in the thin film, which can be used to probe orientation of the dye molecules in the film.

While the AFM micrographs provide inconclusive information as to the structure of the film below the interface, the combination of homogeneous optical images and the polarized absorption data from the thin films reveals the film structure. Polarized microscopy show that the films have a uniform structure, while the polarized spectroscopy shows that the dye molecules are oriented with respect to the surface normal of the substrate. 

By comparing the results from the stretched polymer films with that of the thin films, the polarized absorption data reveals a structure, where the long axis of the dye molecules are either parallel or perpendicular to the surface normal. In analyzing the data, we know that the transition moment **M_f_** is confined to the plane of the molecule. That does not allow for unique determination of the structure, as we are not able to discriminate between the situations where ω_zZ’_ = α_fZ_ − ϕ_fz_ and ω_zZ_ = α_fZ_ + ϕ_fz_ (see Figure 7). If the two situations are considered with regard to the substrate, they both corresponds to a situation where a side of the rectangular dye is parallel to the substrate surface (see Figure 7). This orientation is fully consistent with a lamellar structure. Considering the placement of the charged groups, one orientation is more likely than the other (see Figure 7). It is assumed that the charged groups must be exposed to the surfactant layer, rather than buried in the dye layer. The resulting structure has the shortest side of **AllR** and **Amar** as the repeat distance, while **BorR** has the longer side as the repeat distance. These finding agree with the analysis of structure of the bulk materials, and support that the lamellar structure is present in both bulk materials and thin films.

## 3. Materials and Methods

### 3.1. Synthesis and Characterization

The dyes and the surfactants were all purchased form Sigma-Aldrich, Søborg, Denmark and used directly. Deionized H_2_O was used in the preparation of the complex. The surfactant-dye complex was synthetized by precipitation from an aqueous solution. General procedure: a 2% surfactant solution in deionized water was added to a 2% solution of the dye in the correct ratio as evaluated from the overall charges (1:2, 1:3 or 1:4 respectively). The resulting solution was extracted with dichloromethane, the organic phase washed with water, and dried over magnesium sulfate. The solvent was removed in vacuum, and the resulting products were dried in a vacuum oven at 60 °C. The identity of the materials was confirmed by mass spectrometry (MS) using Electrospray Ionization (ESI) with a Time of Flight detector (TOF). The purity of the compounds was confirmed by elemental analysis performed by Birgitta Kegel at the University of Copenhagen.

All films were prepared by spin-casting via the same method: 20 μL of dichloromethane stock solution (1–5 mg/mL in dichloromethane, methanol was added if the compound did not dissolve) were dropped onto a standard microscope glass slide (Menzel Gläzer, precleaned by washing with water and methanol) spinning at ~2000 rpm. No thermal annealing was applied; all films have been stored at ambient conditions, and investigated as cast. The thin films were characterized with AFM, widefield microscopy and polarized absorption (tilted plate method).

Stretched polymer films were prepared by dissolving the dyes in a 10% PVA solution in water, to a final concentration of 10 µM, gently mixing to avoid bubbles and letting the mixture dry out in Petri Dishes for ca. 1 month. The colored polymer was cut from the dish and placed in a stretcher where it was stretched 5.7 times. 

Powder diffraction was performed on samples that had been crushed using mortar and pistil and compacted in polymer supported sample holders or low volume zero-background sample holders. Several replicas were recorded for each sample and replica of selected samples were recorded in two different sample holders to ensure identical results. The samples were all used directly after the drying procedure, effectively a 60 °C annealing over several days. A Bruker D8 Advance diffractometer was used fitted with suitable optics and a Cu X-ray tube emitting at 1.5418 Å was used for all samples.

Widefield and darkfield microscopy pictures were taken with an Axiocam MRc camera (Zeiss, Birkerød, Denmark) fitted onto a Zeiss axioscope microscope.

All AFM images were recorded with a Veeco Dimension 3000, Aschheim, Germany microscope, and the subsequent data processing were conducted with use of the freeware Gwyddion 2.50 [52]. 

### 3.2. Polarized Spectroscopy

Polarized absorption spectra of the thin films were recorded using a Perkin Elmer Lambda 1050 (Waltham, MA, USA) with a dedicated sample holder which allows control of the incident angle of the light and the use of horizontally polarized light. A clean microscope slide at the same tilt angle as the thin films was used as baseline reference. For the stretched polymers, horizontal and vertical polarization were used and the incident angle was kept at 0°. The blank was acquired using a 10% PVA film stretched 5.7 times.

## 4. Conclusions

Three series of homolog materials were prepared using ionic self-assembly between anionic azo-dyes and benzalkonium surfactants. The lamellar materials were processed into thin films using spin-casting from dichloromethane. The bulk structure of the materials was investigated, and was found to vary throughout the series in the anticipated manner, where the length of the benzalkonium surfactant determines the lamella spacing, although the complicated phase-behavior of the materials merits further study using small angle scattering and calorimetry. 

The thin film structure was investigated using optical microscopy, AFM and polarized spectroscopy. The thin films of all sixteen materials were found to be homogeneous on the macroscopic and microscopic scale. On the nanoscale, the films varied in morphology and only some materials showed a surface structure expected from a thin film with lamellar structure. Therefore, the anticipated systematic variations within the homologous series were not observed in the thin films. We conclude that a more rigorous control of the thermal history and the spin-casting conditions must be in place to ensure that the phase behavior of the materials does not interfere with the structural analysis.

Analysis of a large area of the thin films was performed using polarized spectroscopy. The established methodology of Michl and Thulstrup was updated to be suitable for use with modern numerical methods [32,33]. A method for estimating the angle ϕ_fz_ between the transition moments and the long axes of the molecule from a smaller dataset than used in standard treatment by Michl and Thulstrup was presented. The determined angle ϕ_fz_ was used to translate the average orientation angle determined for the dyes α_fZ,max_ into a proposed molecular structure in the thin films. The polarized absorption experiments and subsequent data analysis confirmed the presence of order in the thin films. We conclude that polarized optical spectroscopy can be a very powerful tool in analyzing the molecular order in thin films, in particular if the orientation of the absorbing transition moment is well-defined within the molecular framework. We showed that polarized optical spectroscopy can give additional information on the molecular structure in the proposed lamellar structure of the thin films, but further studies are required before we can rationalize the lack of response from the films in the polarized optical microscope.

## Figures and Tables

**Table 1:** Method of isolation and elemental analysis results for the 16 synthesized materials.

Entry	Material	H_2_O	C		H		N	
	Allura Red **AllR**		Calcd	Found	Calcd	Found	Calcd	Found
1	**BZK10** precipitated	½	66.44	66.63	8.26	8.42	5.53	5.52
2	**BZK12** precipitated	½	67.44	67.44	8.58	8.83	5.24	5.18
3	**BZK14** extracted	0.00	68.90	69.06	8.85	9.33	5.02	4.77
4	**BZK16** extracted	1.00	67.11	67.02	9.13	9.20	4.74	4.60
5	**BZK18** extracted	0.00	70.43	70.12	9.36	9.37	4.56	4.54
	Bordeaux Red **BorR**							
6	**BZK10** extracted	½	68.40	68.67	8.02	7.28	5.50	5.55
7	**BZK12** precipitated	0.00	69.90	69.61	8.33	8.30	5.26	5.25
8	**BZK14** extracted	0.00	69.70	69.75	8.91	8.66	5.16	4.96
9	**BZK16** extracted	wet^2^	-	61.24	-	9.24	-	3.64
10	**BZK18** extracted	4.00	68.06	68.31	9.26	9.32	4.29	4.11
	Amaranth **Amar**							
11	**BZK10** extracted	2.00	66.01	65.87	8.42	8.28	5.00	4.91
12	**BZK12** extracted	½	68.37	68.29	8.71	9.28	4.80	4.60
13	**BZK14** extracted	½	69.31	69.24	9.02	9.01	4.54	4.45
14	**BZK16** extracted	2.00	69.01	69.29	9.33	9.82 ^1^	4.24	4.12
15	**BZK18** precipitated	0.00	71.23	71.06	9.54	10.30 ^1^	4.12	3.95
	Trypan Blue **TryB**							
16	**BZK10** precipitated	1.00	66.96	66.86	8.13	8.32	7.10	6.83

^1^ Experimental error too large to be physical, data not used; ^2^ Material composition unknown and the results are not used in the subsequent analysis.

**Table 2:** Structural information from polarized optical spectroscopy on thin films of ionic self-assembled material based on Allura Red, Bordeaux red and Amaranth anionic azo-dyes and benzalkonium surfactants with alkane tail of varying length (from –(CH_2_)_9_CH_3_ to –(CH_2_)_17_CH_3_): The average angle (respect to the surface normal) of the transition moment corresponding to primary transition α_fZ,max_ and maximum of primary transition *λ*_max_. Direct structural information from: terrace heights *d’* determined from AFM micrographs of the same thin films, and layer separation *d* determined in bulk powder using XRD. The difference in layer spacing ∆*d* was calculated using the XRD data.

Entry	Material	α_fZ,max_ *^a^* (deg)	*λ*_max_ (nm)	*d*_XRD_ (nm)	∆*d*	*d’*_AFM_ (nm)
Allura red **AllR**						
1	**BZK10**	54.4	500	4.42	-	-
2	**BZK12**	53.2	500	4.71	0.3	2.65
3	**BZK14**	55.9	500	-	-	5.85
4	**BZK16**	60.1	500	-	-	2.5&1.5
5	**BZK18**	63.2	500	5.75	-	2.8–3.2
Bordeaux red **BorR**						
6	**BZK10**	62.1	510	3.63	-	2.8 & 2.2
7	**BZK12**	63.9	510	3.87	0.2	-
8	**BZK14**	61.4	510	4.06	0.2	-
9	**BZK16**	59.9	510	-	-	-
10	**BZK18**	55.2	510	4.66	0.2	2.5–3.3
Amaranth **Amar**						
11	**BZK10**	64.9	520	3.72	-	7.5–8.0
12	**BZK12**	62.2	520	4.18	0.4	-
13	**BZK14**	63.3	520	4.38	0.2	2.1
14	**BZK16**	64.8	520	4.96	0.6	3.9
15	**BZK18**	63.2	520	5.18	0.2	2.6 & 3.7
Trypan Blue **TrypB**						
16	**BZK10**	80	617	2.51	-	-

*^a^* Values determined using a value of the refractive index of the thin films of *n* = 1.45.

**Figure A1:**
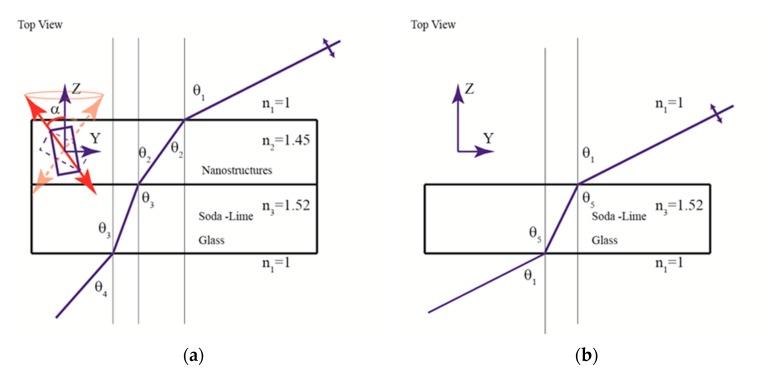
Sketch of the tilted plate setup for the thin films: (**a**) sample with spin casted nanostructures on glass; and (**b**) glass blank sample.

## Supplementary Materials

The following are available online at http://www.mdpi.com/2079-4991/8/2/109/s1, File S1.pdf: Supporting information including AFM, microscopy and polarized absorption data from all 16 materials, File S2.zip: MatLab functions for analyzing the polarized absorption data and plotting the results.

## Appendix A. Theory Used in Polarized Spectroscopy

The data analysis follows the theory developed for oriented molecular systems by Thulstrup and Michl and Graf et al. [40,41] The theory applied to the investigated systems is re-iterated and adapted below, and an explanation of the assumptions is also given. The scripts used in the analysis are part of the Supplementary Materials.

### Appendix A.1 Stretched Polymers

The aim of this section is to relate the angle $\alpha_{\mathrm{fZ}}$ between the long axis z of the molecular framework and the transition moment **M_f_** with the experimental variables. In this experiment, we use a different approach from that of Thustrup and Michl. The most general case involves finding the orientation factors after measuring five differently polarized transitions and subsequently finding the average angle of a certain molecular axes with respect to the transition moment upon certain assumptions. Here, the orientation factors were not measured, rather the spread of the value of the angle of interest was analyzed as a function of all the possible values of the orientation factors allowed by our assumptions on the molecules (see Figure 4 and Figure 5) and data from one transition.

The essential elements of the setup are sketched in 2D in Figure 4d.

The average squared direction cosine of the transition moment **M_f_** with respect to the stretch direction *Z* is denoted $k_{f}$ and given by [41]

(A1) $$k_{f}=\cos^{2}\left(M_{f},\;Z\right)$$

The dichroic ratio for a non-overlapped transition is defined as [41]

(A2) df=EZEY

where $E_{Z}$ and $E_{Y}$ are the measured optical densities for horizontal and vertical polarized incident light, respectively, corrected with a blank sample and corrected for additional scattering. Assuming uniaxial alignment with respect to the stretch direction *Z*, the dichroic ratio is related to the direction cosine of the transition by [41]

(A3) $$k_{f}=\frac{d_{f}}{2+d_{f}}$$

The angle $\alpha_{\mathrm{fz}}$ is the average angle of the transition moment **M_f_** with respect to the *Z* axes, and can be found from

(A4) $$\alpha_{\mathrm{fZ}}=\arccos\left(\sqrt{k_{f}}\right)$$

The angle $\phi_{fz}$ is calculated from the measured $k_{f}$ under two different assumptions of orientation factors of the molecules in the thin-film: rod-shape molecules or planar molecules. In both cases, the angles satisfy the same equation [40]

(A5) $$\tan^{2}\phi_{\mathrm{fz}}=\frac{k_{z}-k_{f}}{k_{f}-k_{y}}$$

where $k_{z}$ and $k_{y}$ are the orientation factors of the molecular framework axes, i.e., the average squared direction cosines of the molecular axes z and y with respect to the stretch direction Z. The orientation factor is directly related to the shape of the molecule, although it is not the only factor determining molecular orientation, as noted by Thulstrup and Michl [40]. However, we shall refer to molecular shape and orientation indistinctly as they did in their work. The orientation factors satisfy

(A6) $$k_{x}+k_{y}+k_{z}=1$$

Additionally, only the values of the orientation factors inside the orientation triangle (Figure 4b) are considered [40], wherein $k_{z}\geq k_{y}\geq k_{x}$ is satisfied. In the case of rod-shaped molecules, the orientation factors satisfy [40]

(A7) $$k_{y}=k_{x}=\frac{\left(1-k_{z}\right)}{2}$$

Planar molecules are those which transition moment of interest lies in the plane defined by two molecular axes, in our case assumed to be the longer axes z and y, as this is the common case of π–π* transitions in planar molecules of symmetry C_s_ or C_2h_. Planar molecules are only required to be inside the orientation triangle and satisfy Equation (A5). The limits of the orientation triangle are given by the extreme shapes of the molecule, namely rod-like for the lower left side of the triangle, disk-shaped for the lower right side of the triangle, and “flat” when the *yZ* plane is parallel to the laboratory *Z* axes (in other words, the average projection of the shortest axes of the molecule x with the *Z* axes is always zero). For flat molecules, the orientation factors are given by

(A8) $$k_{y}=\left(1-k_{z}\right);{\;k}_{x}=0$$

To evaluate the angles, the order parameters are evaluated in the domain inside the orientation triangle where $\tan^{2}\phi_{\mathrm{fz}}>0$ is deduced from Equation (A5). This implies the condition $k_{z}\geq k_{f}>k_{y}$ or $k_{z}>k_{f}\geq k_{y}$, which together with the rod-shape line and the “flat” line limit the possible values inside the orientation triangle.

### Appendix A.2. Thin Film Data

The aim of this section is to relate the angle $\alpha_{\mathrm{fZ}}$ of the transition moment $M_{f}$ with respect to the *Z* axes with the experimental variables. In this case, the molecules are uniaxially distributed around the axes *Z* normal to the substrate plane. A cartoon of the setup is shown in Figure A1.

The absorption coefficient of the nanostructures $E_{N}$ can be written as a sum of a component produced by the axis normal to the surface of the thin film *Z* and by an axis parallel to the surface Y [41,43].

(A9) $$E_{N}=E_{Y}\cos^{2}\theta_{2}+E_{Z}\sin^{2}\theta_{2}$$

where $\theta_{2}$ is the angle of the light travelling through the nanostructures with respect to the Z axis. A relation of $E_{Y},{\;E}_{Z}$ to the susceptibilities can be found in Graf et al. [43]. The wavelength dependences of the absorption coefficient E, optical density OD, refractive index n, and transmittance T are not explicitly written for brevity.

By rewriting Equation (A9), $E_{Z}$ and $E_{Y}$ can be found from the linear regression of $E_{N}$ against $\cos^{2}\theta_{2}$ with

(A10) $$E_{N}=\left(E_{Y}-E_{Z}\right)\cos^{2}\theta_{2}+E_{Z}$$

The quantities $E_{Z}$ and $E_{Y}$ are directly related to $\alpha_{\mathrm{fZ}}$, by Equations (A2)–(A4). The order parameter $A_{2}$, which is described later in detail, can also be extracted from this regression and it is related to $\alpha_{\mathrm{fZ}}$ [43].

Now, we want to deduce the quantities $E_{N}$ and $\theta_{2}$ from our measured optical density and the known incident angle $\theta_{1}$. From Snell’s law we can find the relation of ${\cos\theta }_{2}$ with our experimental parameter $\theta_{1}$

(A11) $${\cos\theta }_{2}=\sqrt{1-{\left(\frac{n_{1}}{n_{2}}\sin\theta_{1}\right)}^{2}}$$

In a slightly modified version of the formalism used by Graf et al., we can extract the absorption coefficient of the nanostructures using the background measurement. Here, the absorption coefficient is noted as E to avoid confusion with the angle α. Note that in the stretched polymer section, the variable E denotes the corrected optical density instead of the absorption coefficient, since it is not possible to analyze the transmittance in the same manner as here. For the sample in Figure A1a consisting of nanostructures spin-casted on glass, we can deduce that the measured optical density OD satisfies:

(A12) ODNG=−log10TNG+(ENdNcosθ2+EGdGcosθ3)log10e

where N denotes nanostructures and G glass, d is the thickness of the different layers, θ is the angle of the light with respect to the normal of the sample through the different layers (calculated with Snell’s law) and T is the transmittance derived from Fresnel equations for *p*-wave. The transmittance is the product of the transmittance through the different layers:

(A13) $$T_{NG}=T_{A\to N}T_{N\to G}T_{G\to A}$$

where the index A stands for air. As an example, the transmittance $T_{N\to G}$ is

(A14) $$T_{N\to G}\left(n_{N},n_{G},\theta_{2},\theta_{3}\right)=1-{\left|\frac{n_{N}\cos\theta_{3}-n_{G}\cos\theta_{2}}{n_{N}\cos\theta_{3}+n_{G}\cos\theta_{2}}\right|}^{2}$$

The other transmittances can be equally evaluated with the dependencies $T_{A\to N}\left(n_{A},n_{N},\theta_{1},\theta_{2}\right)$ and $T_{G\to A}\left(n_{G},n_{A},\theta_{3},\theta_{4}\right)$.

From the measured blank sketched in Figure A1b, we can deduce a similar equation for the optical density

(A15) ODG=−log10TG+(EGdGcosθ5)log10e

where in this case the transmittance reads

(A16) $$T_{G}=T_{A\to G}{T^{\prime }}_{G\to A}$$

In this case, the dependencies are $T_{A\to G}\left(n_{A},n_{G},\theta_{1},\theta_{5}\right)$ and ${T^{\prime }}_{G\to A}\left(n_{G},n_{A},\theta_{5},\theta_{1}\right)$.

Combining Equations (A12) and (A15), we can eliminate the dependence on $E_{G}d_{G}$ and derive the absorption coefficient of the nanostructures

(A17) $$E_{N}=\left[\left({OD}_{NG}+\log_{10}T_{\mathrm{NG}}\right)-\left({OD}_{G}+\log_{10}T_{G}\right)\frac{\cos\theta_{5}}{\cos\theta_{3}}\right]\frac{\cos\theta_{2}}{d_{s}\log_{10}e}$$

To find the angle $\alpha_{\mathrm{fZ}}$ and the order parameter $A_{2}$, it is not necessary to know the thickness of the sample as the term $d_{s}\log_{10}e$ cancels out. Strictly, the regression is performed with the quantity ${kE}_{N}$ against $\cos^{2}\theta_{2}$, where $k=d_{s}\log_{10}e$, by using the equation

(A18) $${kE}_{N}=k\left(\left(E_{Y}-E_{Z}\right)\cos^{2}\theta_{2}+E_{Z}\right)$$

from which the quantities of interest can be derived from the slope a and the intercept b. Using the same definition as in Equation (A2), the dichroic ratio can be found as $d_{f}=\frac{b}{a+b}$, from which the angle $\alpha_{\mathrm{fZ}}$ can be calculated using Equations (A3) and (A4) as:

(A19) $$\alpha_{\mathrm{fZ}}=\arccos\left(\sqrt{\frac{\left(\frac{b}{a+b}\right)}{2+\left(\frac{b}{a+b}\right)}}\right)$$

The order parameter from Graf et al., $A_{2}$, can be found in [43].

Here, this quantity is only calculated at the wavelength corresponding to the emission maxima, where we use the value of the refractive index of azobenzene $n_{2}=1.45$, and the value $n_{3}=1.52$ for Borosilicate glass [53]. An example of the application of this procedure is shown in Figure 6.
