# Supplementary material for: Using Polarized Spectroscopy to Investigate Order in Thin-Films of Ionic Self-Assembled Materials Based on Azo-Dyes
Source: Nanomaterials (Basel). 2018 Feb 15;8(2):109. doi: 10.3390/nano8020109 (PMC5853740; doi:10.3390/nano8020109)
Supplement: Supplementary file 1 [file nanomaterials-08-00109-s001.pdf]

nanomaterials

MDPI

Supplementary Materials

Using Polarized Spectroscopy to Investigate Order in Thin-Films of Ionic Self-Assembled Materials Based on Azo-Dyes

Miguel R. Carro-Temboury,* Martin Kühnel, Mariam Ahmad, Frederik Andersen, Ári Brend Bech, H. Krestian L. Bendixen, Patrick R. Nawrocki, Anders J. Bloch, Ilkay Bora, Tahreem A. Bukhari, Nicolai V. Bærentsen, Jens Carstensen, Smeeah Chima, Helene Colberg, Rasmus T. Dahm, Joshua A. Daniels, Nermin Dinckan, Mohamed El Idrissi, Ricci Erlandsen, Marc Førster, Yasmin Ghauri, Mikkel Gold, Andreas Hansen, Kenn Hansen, Mathias Helmsøe-Zinck, Mathias Henriksen, Sophus V. Hoffmann, Louise O. H. Hyllested, Casper Jensen, Amalie S. Kallenbach, Kirandip Kaur, Suheb R. Khan, Emil T. S. Kjær, Bjørn Kristiansen, Sylvester Langvad, Philip M. Lund, Chastine F. Munk, Theis Møller, Ola M. Z. Nehme, Mathilde Rove Nejrup, Louise Nexø, Simon Skødt Holm Nielsen, Nicolai Niemeier, Lasse V. Nikolajsen, Peter C. T. Nøhr, Dominik B. Orlowski, Marc Overgaard, Jacob Skaarup Ovesen, Lucas Paustian, Adam S. Pedersen, Mathias K. Petersen, Camilla M. Poulsen, Louis Praeger-Jahnsen, L. Sonia Qureshi, Nicolai Ree, Louise S. Schiermacher, Martin B. Simris, Gorm Smith, Heidi N. Smith, Alexander K. Sonne, Marko R. Zenulovic, Alma Winther Sørensen, Karina Sørensen, Emil Vogt, Andreas Væring, Jonas Westermann, Sevin B. Özcan and Thomas Just Sørensen *

Nano-Science Center & Department of Chemistry, University of Copenhagen, Universitetsparken 5, 2100 København Ø, Denmark; martin.kuhnel@nano.ku.dk (M.K.); mah@science.ku.dk (M.A.); zgb268@alumni.ku.dk (F.A.); dfb556@alumni.ku.dk (Á.B.B.); skf209@alumni.ku.dk (H.K.L.B.); tbu@science.ku.dk (P.R.N.); gcf945@alumni.ku.dk (A.J.B.); ilkay.bora@gmail.com (I.B.); xrs483@alumni.ku.dk (T.A.B.); gtw317@alumni.ku.dk (N.V.B.); cgm648@alumni.ku.dk (J.C.); mnb838@alumni.ku.dk (S.C.); wkj412@alumni.ku.dk (H.C.); lvf878@alumni.ku.dk (R.T.D.); tqn207@ku.dk (J.A.D.); jdq431@alumni.ku.dk (N.D.); lnk170@alumni.ku.dk (M.E.I.); dgk483@alumni.ku.dk (R.E.); dhm812@alumni.ku.dk (M.F.); wqn556@alumni.ku.dk (Y.G.); fbm685@alumni.ku.dk (M.G.); wjg795@alumni.ku.dk (A.H.); rjf884@alumni.ku.dk (K.H.); bpg317@alumni.ku.dk (M.H.-Z.); tqn207@alumni.ku.dk (M.H.); vxk757@alumni.ku.dk (S.V.H.); rsz113@alumni.ku.dk (L.O.H.H.); pxv130@alumni.ku.dk (C.J.); smh645@alumni.ku.dk (A.S.K.); nhs827@alumni.ku.dk (K.K.); qwm886@alumni.ku.dk (S.R.K.); sgz864@alumni.ku.dk (E.T.S.K.); vtz390@alumni.ku.dk (B.K.); cts952@alumni.ku.dk (S.L.); sqv821@alumni.ku.dk (P.M.L.); chastine.munk@ind.ku.dk (C.F.M.); jzg995@alumni.ku.dk (T.M.); dtw667@alumni.ku.dk (O.M.Z.N.); bnw543@alumni.ku.dk (M.R.N.); gtf274@alumni.ku.dk (L.N.); djq495@alumni.ku.dk (S.S.H.N.); zvl379@alumni.ku.dk (N.N.); fwn674@alumni.ku.dk (L.V.N.); hgv633@alumni.ku.dk (P.C.T.N.); mnc612@alumni.ku.dk (D.B.O.); marc.overgaard@nano.ku.dk (M.O.); kvn491@alumni.ku.dk (J.S.O.); cfh645@alumni.ku.dk (L.P.); zwx958@alumni.ku.dk (A.S.P.); scd880@alumni.ku.dk (M.K.P.); bcr102@alumni.ku.dk (C.M.P.); bvh517@alumni.ku.dk (L.P.-J.); ptr511@alumni.ku.dk (L.S.Q.); cmh133@alumni.ku.dk (N.R.); nwr275@alumni.ku.dk (L.S.S.); dsk326@ku.dk (M.B.S.); dsk326@alumni.ku.dk (G.S.); cfh645@alumni.ku.dk (H.N.S.); zwx958@alumni.ku.dk (A.K.S.); markozenulovic@gmail.com (M.R.Z.); alma.soerensen@sund.ku.dk (A.W.S.); marc.overgaard@chem.ku.dk (K.S.); bvh517@ku.dk (E.V.); qwm886@ku.dk (A.V.); jdq431@ku.dk (J.W.); sgz864@ku.dk (S.B.Ö.)

* Correspondence: miguel@chem.ku.dk (M.R.C.-T.); tjs@chem.ku.dk (T.J.S.); Tel.: +45-3532-0213 (T.J.S.)

nanomaterials

Table of contents

Author Contributions **3**

Data Analysis **4**

Allura red BZK10 Hold 14 5

Allura red BZK12 Hold 15 6

Allura red BZK14 Hold 9 7

Allura red BZK16 Hold 4 8

Allura red BZK18 Hold 10 10

Bordeaux red BZK10 Hold 12 11

Bordeaux red BZK12 Hold 13 12

Bordeaux red BZK14 Hold 8 13

Bordeaux red BZK16 Hold 11 14

Bordeaux red BZK18 Hold 16 15

Amaranth BZK10 Hold 3 17

Amaranth BZK12 Hold 1 19

Amaranth BZK14 Hold 6 20

Amaranth BZK16 Hold 5 21

Amaranth BZK18 Hold 2 22

Trypan Blue BZK10 Hold 7 24

Polarised Spectroscopy **26**

Stretched polymer analysis **28**

Author Contributions

Miguel R. Carro-Temboury designed the polarized optical experiments and the data analysis template, validated data analysis, and assisted in the writing of the paper.

Martin Kühnel recorded and helped analyzing the AFM micrographs, validated data analysis, and assisted in the writing of the paper.

Mariam Ahmad, Frederik Andersen, Ári B. Bech, H. Krestian L. Bendixen, Patrick R. N. Blichfeldt, Anders J.Bloch, Tahreem A. Bukhari, Nicolai V.Bærentsen, Jens Carstensen, Smeeah Chima, Helene Colberg, Rasmus T. Dahm, Joshua A.Daniels, Nermin Dinckan, Mohamed El Idrissi, Ricci F.Erlandsen, Marc Førster, Yasmin Ghauri, Mikkel Gold, Andreas Hansen, Kenn Hansen, Mathias Helmsøe-Zinck, Mathias Henriksen, Sophus V. Hoffmann, Louise O. H. Hyllested, Casper Jensen, Amalie S. Kallenbach, Kirandip Kaur, Suheb R. Khan, Emil T. S. Kjær, Bjørn Kristiansen, Sylvester H. Langvad, Philip M. Lund, Chastine F. Munk, Theis Møller, Ola M. Z. Nehme, Mathilde R. Nejrup, Louise Nexø, Simon Skødt Holm Nielsen, Nicolai Niemeier, Lasse V. Nikolajsen, Peter C. T. Nøhr, Dominik B. Orlowski, Jacob S. Ovesen, Lucas Paustian, Adam S. Pedersen, Mathias K. Petersen, Camilla M. Poulsen, Louis Praeger-Jahnsen, L. Sonia Qureshi, Nicolai Ree, Louise S. Schiermacher, Martin B. Simris, Gorm Smith, Heidi N. Smith, Alexander K. Sonne, Marko R. Zenulovic, Alma L. W. Sørensen, Karina Sørensen, Emil Vogt, Andreas Væring, Jonas Westermann, Sevin B. Özcan, designed the experiment, synthesized, purified and characterized the investigated materials. Prepared thin films, XRD powder samples and investigated the former using optical microscopy and AFM. Analysed the composition of the compounds, analyzed the data from polarized spectroscopy and interpreted the structure related data. Read and commented on/corrected the final manuscript.

Ilkay Bora supervised the synthesis and characterisation of the materials, validated elemental analysis and mass spectrometry data, and read and commented on the final draft of the manuscript.

Marc H. Overgaard helped/recorded optical microscopy images. Read and commented on the final manuscript.

Thomas Just Sørensen designed the experiments, helped in the synthesis and data analysis. Compiled the supporting information and wrote the paper.

Data Analysis

Scripts for the programs used for data analysis are supplied as part of the supporting information. Three scripts are supplied.

- Nano1_StretchedPolymers.m
- Nano1_ThinFilmAngles.m
- Nano1_OrientationTriangle.m

The data is loaded as x: wavelength in nm; y: absorbance from e.g. CSV files in the first two programs in order to determine ϕ_{fz} and α_{fz} (see main text).

The last program plots the orientation triangles used in the main text Figure 4b and Figure S84–85.

Allura red BZK10 Hold 14

Optical Microscopy

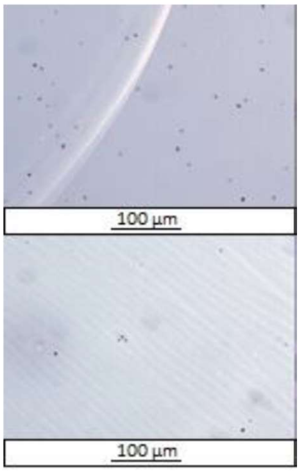

Figure S1. Wide field optical microscopy images of the edge (top) and a central region (bottom) of a thin film.

Atomic Force Microscopy

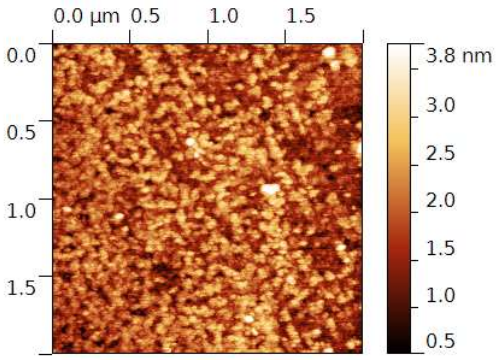

Figure S2. Atomic force microscopy (AFM) micrograph of the central region of a thin film. Distances determined from AFM; No distinct heights found.

Polarised Spectroscopy

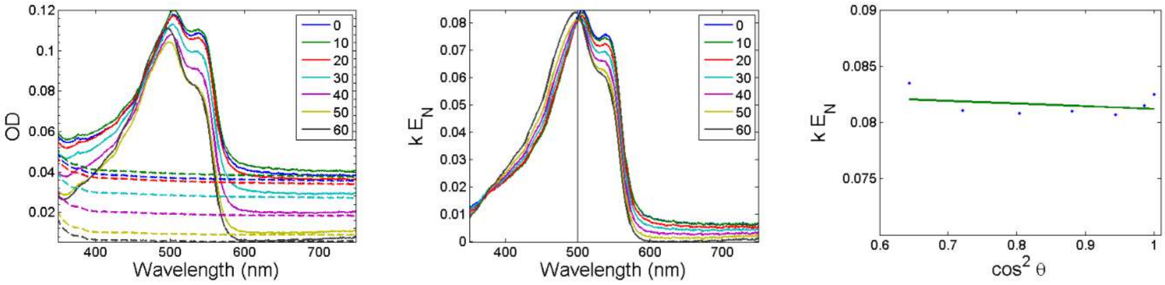

(**A**) (**B**) (**C**)

Figure S3. (**A**) Raw data from polarized optical spectroscopy (tilt angles from 0° to 60° in steps of 10). The spin coated films are represented with solid lines and the background from the glass with dashed lines. (**B**) Corrected spectra given by the constant k times the absorption coefficient E_N. (**C**) Linear regression at the maximum of the emission to find α_{fz}.

nanomaterials

Allura red BZK12 Hold 15

Optical Microscopy

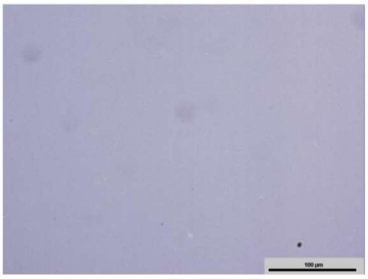

Figure S4. Wide field optical microscopy image of the central region of a thin film.

Atomic Force Microscopy

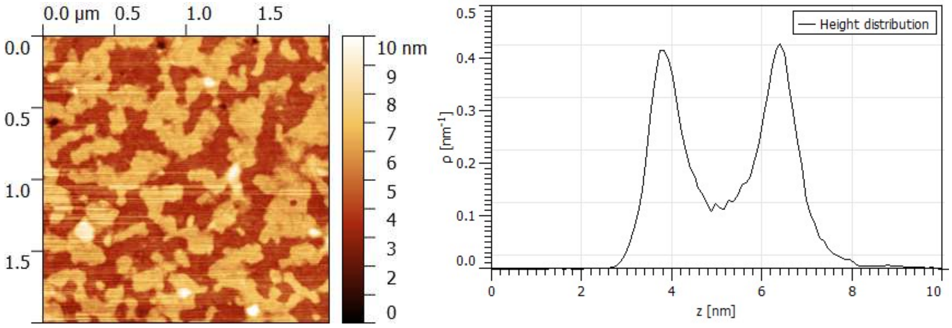

Figure S5. AFM micrograph and height distribution (entire image) from the central region of a thin film.

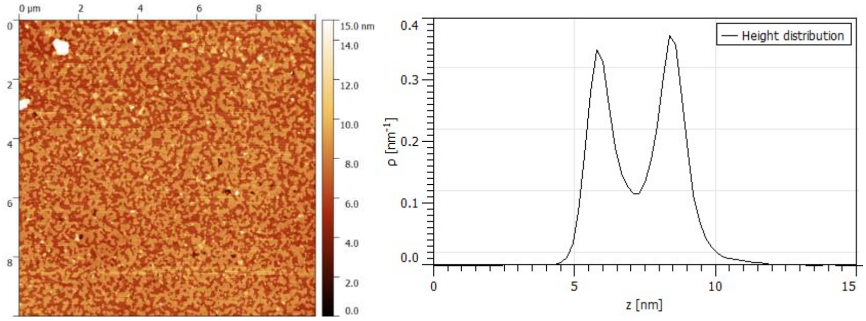

Figure S6. AFM micrograph and height distribution (entire image) from the central region of a thin film.

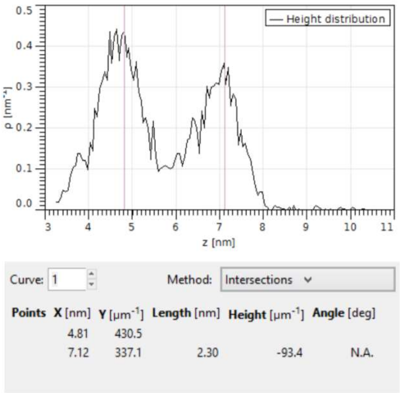

Figure S7. Height distribution taken from a local area in the image given in Figure S6.

Polarized Spectroscopy

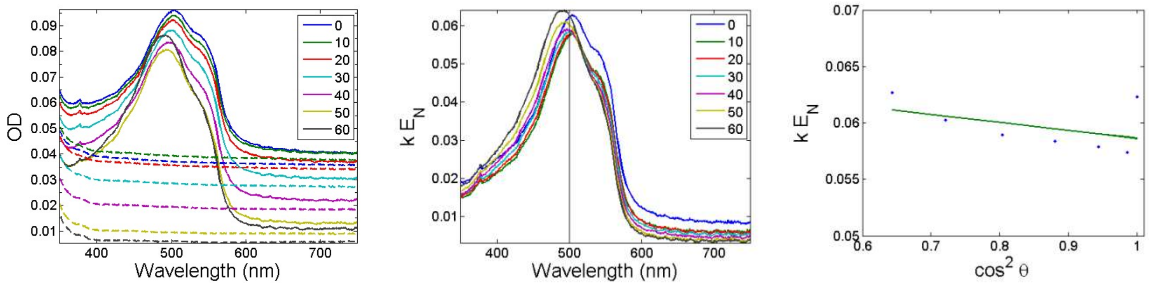

Figure S8. (**Left**) Raw data from polarized optical spectroscopy (tilt angles from 0° to 60° in steps of 10). The spin coated films are represented with solid lines and the background from the glass with dashed lines. (**Center**) Corrected spectra given by the constant k times the absorption coefficient E_N. (**Right**) Linear regression at the maximum of the emission to find α_{fZ}.

Allura red BZK14 Hold 9

Optical Microscopy

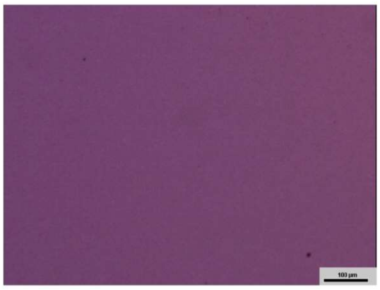

Figure S9. Wide field optical microscopy image of the central region of a thin film.

Atomic Force Microscopy

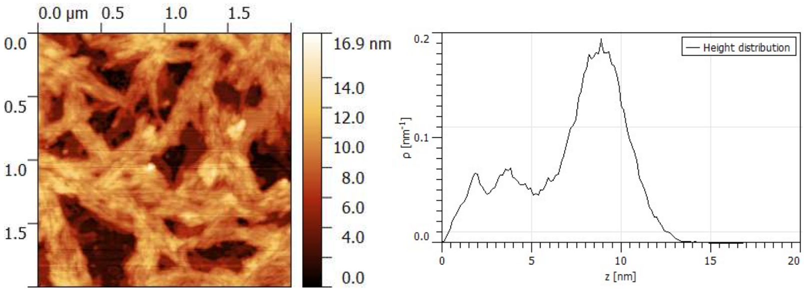

Figure S10. AFM micrograph and height distribution (entire image) from the central region of a thin film.

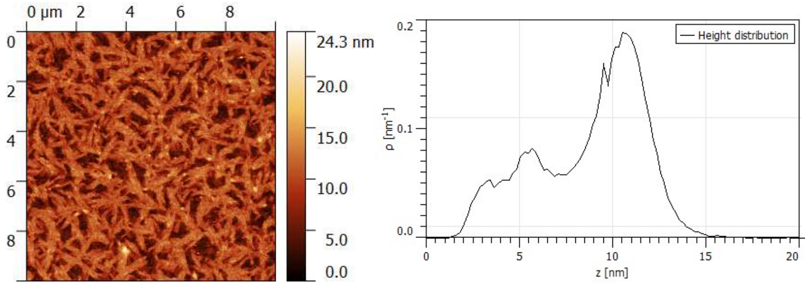

Figure S11. AFM micrograph and height distribution (entire image) from the central region of a thin film.

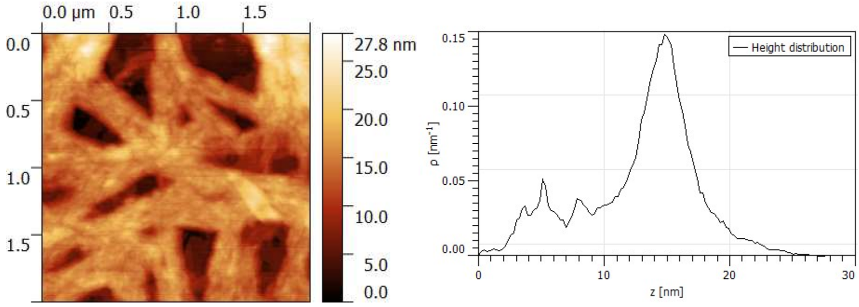

Figure S12. AFM micrograph and height distribution (entire image) from the central region of a thin film.

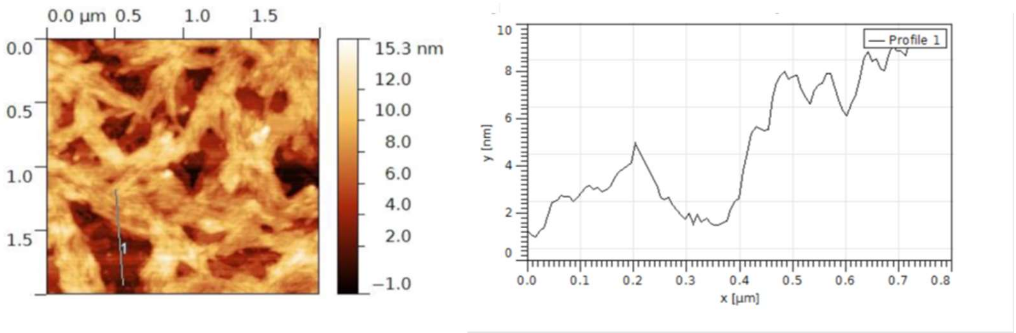

Figure S13. AFM micrograph and height profile (see line in image) from the central region of a thin film.

Polarized Spectroscopy

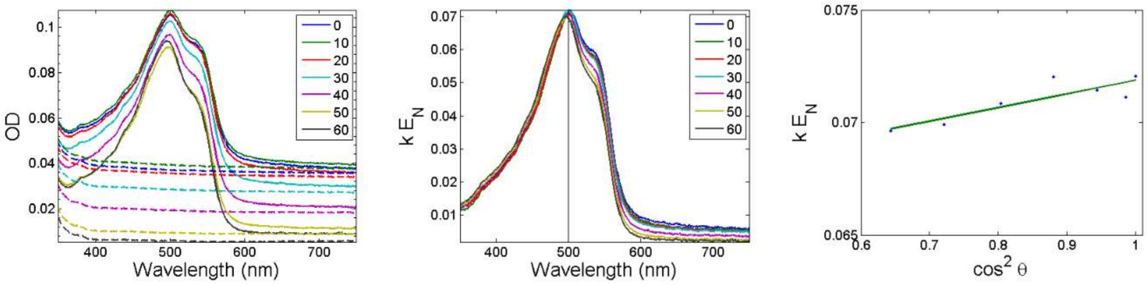

Figure S14. (**Left**) Raw data from polarized optical spectroscopy (tilt angles from 0° to 60° in steps of 10). The spin coated films are represented with solid lines and the background from the glass with dashed lines. (**Center**) Corrected spectra given by the constant k times the absorption coefficient E_N. (**Right**) Linear regression at the maximum of the emission to find α_{fz}.

Allura red BZK16 Hold 4

Optical Microscopy

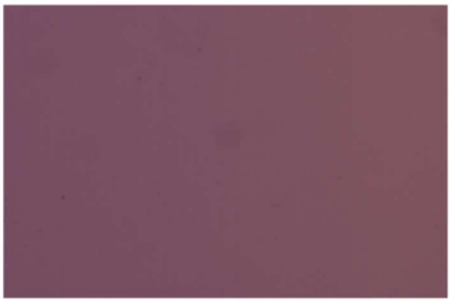

Figure S15. Wide field optical microscopy image of the central region of a thin film

Atomic Force Microscopy.

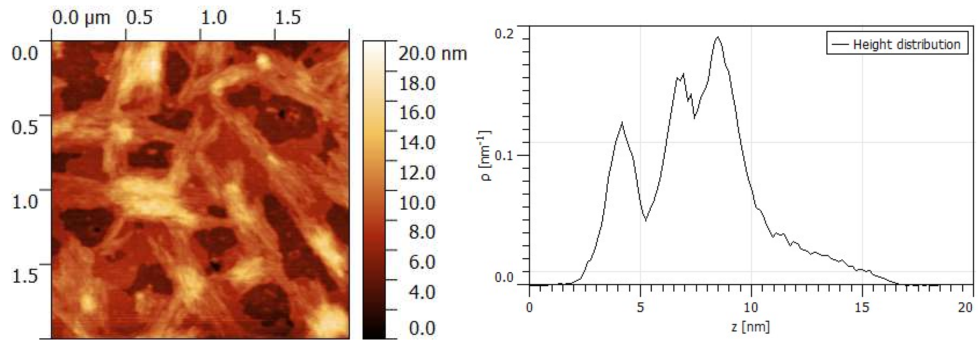

Figure S16. AFM micrograph and height distribution (entire image) from the central region of a thin film.

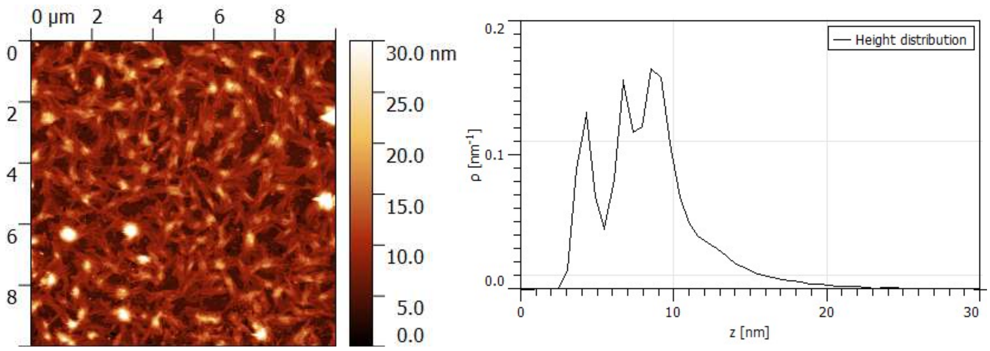

Figure S17. AFM micrograph and height distribution (entire image) from the central region of a thin film.

Polarized Spectroscopy

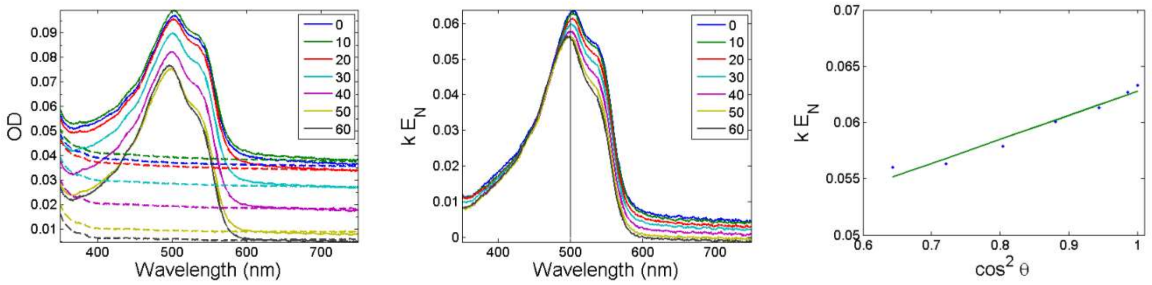

Figure S18. (**Left**) Raw data from polarized optical spectroscopy (tilt angles from 0° to 60° in steps of 10). The spin coated films are represented with solid lines and the background from the glass with dashed lines. (**Center**) Corrected spectra given by the constant k times the absorption coefficient E_N. (**Right**) Linear regression at the maximum of the emission to find α_{fZ}.

nanomaterials

Allura red BZK18 Hold 10

Optical Microscopy

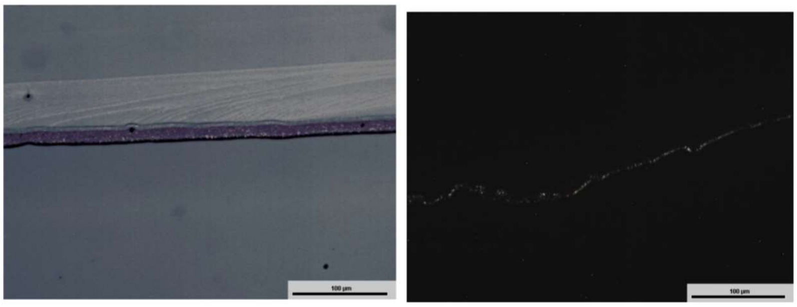

Figure S19. Wide field optical microscopy (top) and dark field microscopy (bottom) images of the edge of a thin film.

Atomic Force Microscopy

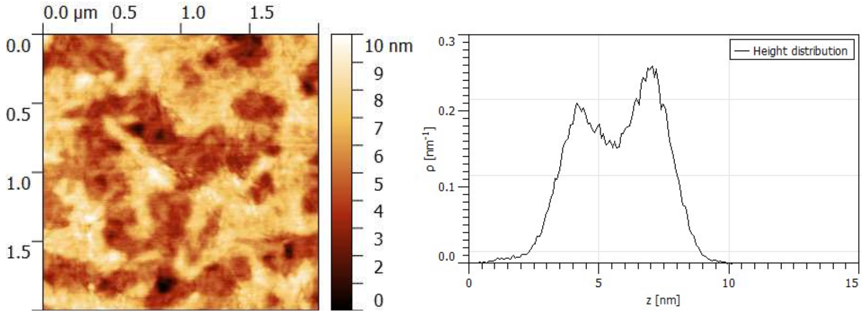

Figure S20. AFM micrograph and height distribution (entire image) from the central region of a thin film.

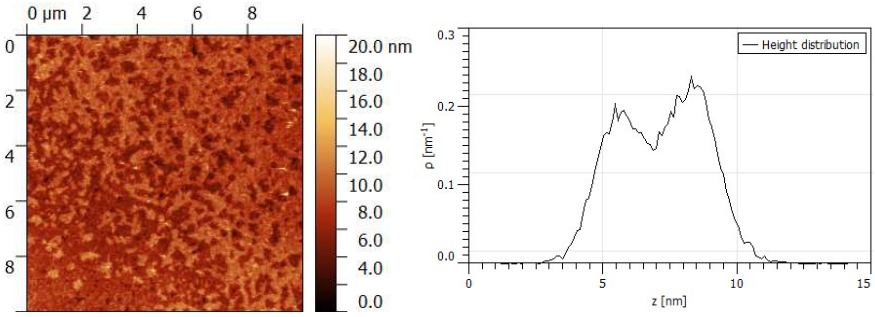

Figure S21. AFM micrograph and height distribution (entire image) from the central region of a thin film.

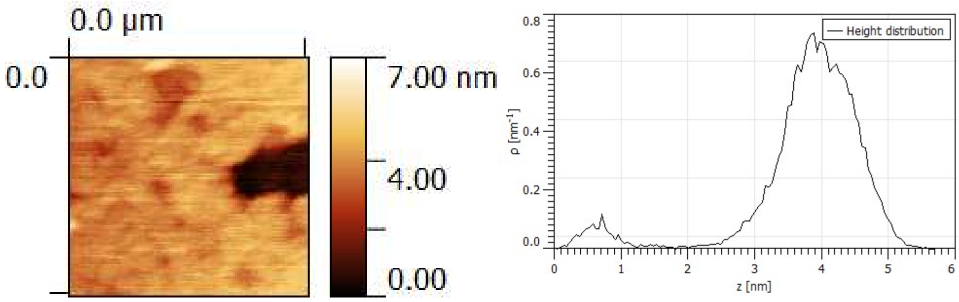

Figure S22. AFM micrograph and height distribution (entire image) from the central region of a thin film.

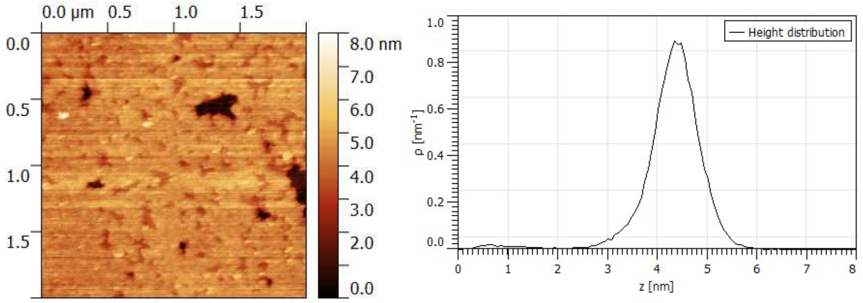

Figure S23. AFM micrograph and height distribution (entire image) from the central region of a thin film.

Polarized Spectroscopy

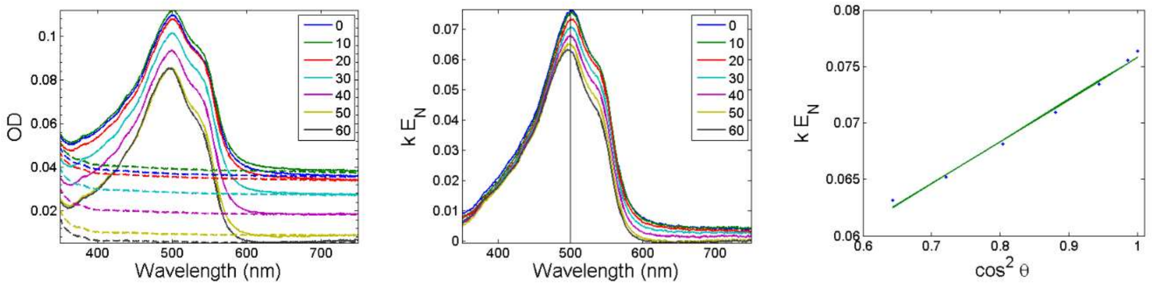

Figure S24. Left) Raw data from polarized optical spectroscopy (tilt angles from 0° to 60° in steps of 10). The spin coated films are represented with solid lines and the background from the glass with dashed lines. Center) Corrected spectra given by the constant k times the absorption coefficient E_N. Right) Linear regression at the maximum of the emission to find α_{fZ}.

Bordeaux red BZK10 Hold 12

Optical Microscopy

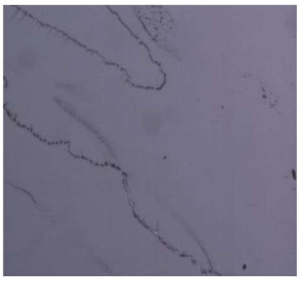

Figure S25. Wide field optical microscopy image of the edge of a thin film.

Atomic Force Microscopy

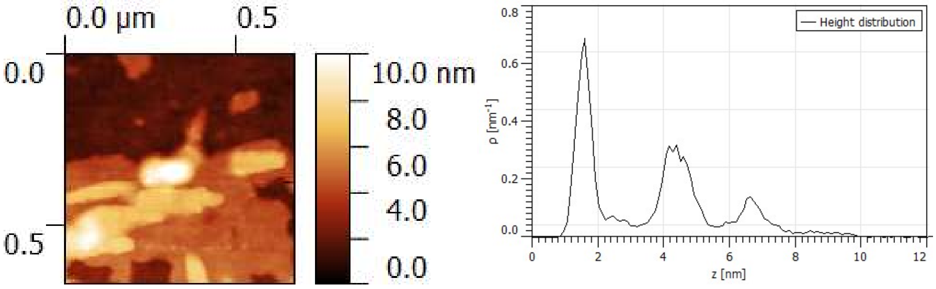

Figure S26. AFM micrograph and height distribution (entire image) from the central region of a thin film.

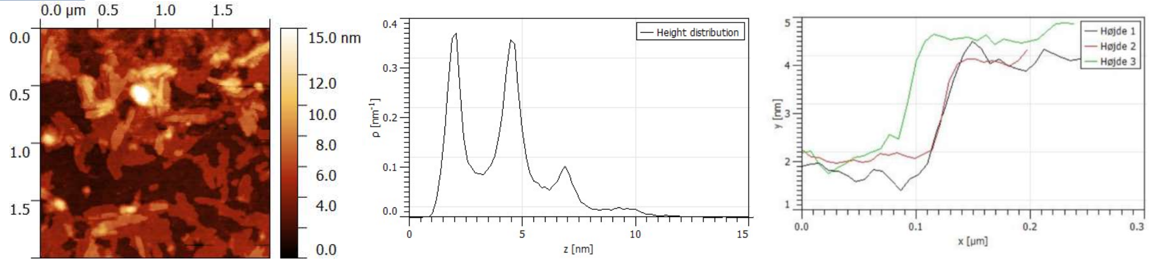

Figure S27. AFM micrograph and height distribution (entire image), and height profiles from the central region of a thin film.

Polarized Spectroscopy

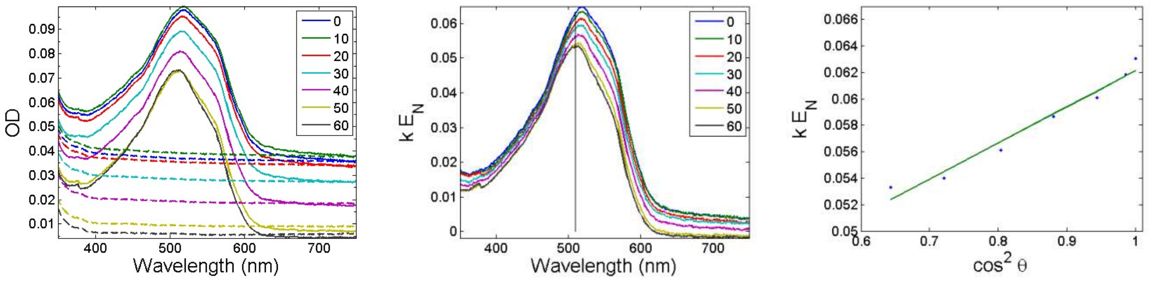

Figure S28. Left) Raw data from polarized optical spectroscopy (tilt angles from 0° to 60° in steps of 10). The spin coated films are represented with solid lines and the background from the glass with dashed lines. Center) Corrected spectra given by the constant k times the absorption coefficient E_N. Right) Linear regression at the maximum of the emission to find α_{fZ}.

Bordeaux red BZK12 Hold 13

Optical Microscopy

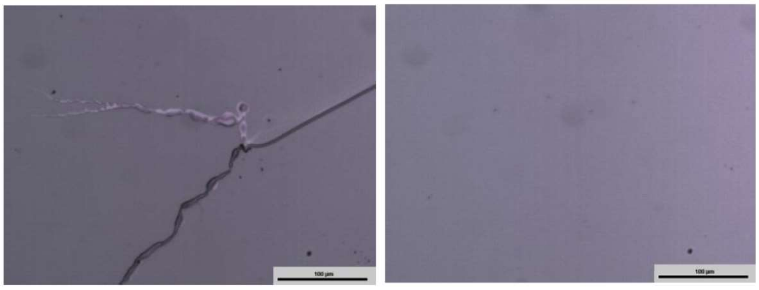

Figure S29. Wide field optical microscopy images of the edge (top) and central region (bottom) of a thin film.

Atomic Force Microscopy

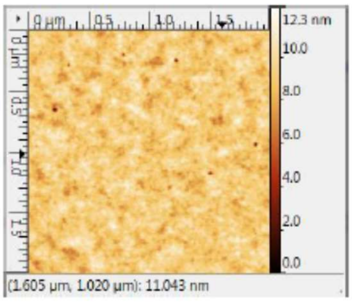

Figure S30. AFM micrograph from the central region of a thin film.

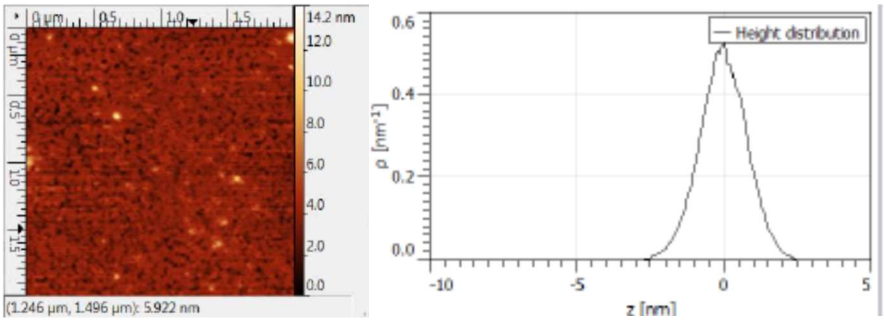

Figure S31. AFM micrograph and height distribution (entire image) from the central region of a thin film.

Polarized Spectroscopy

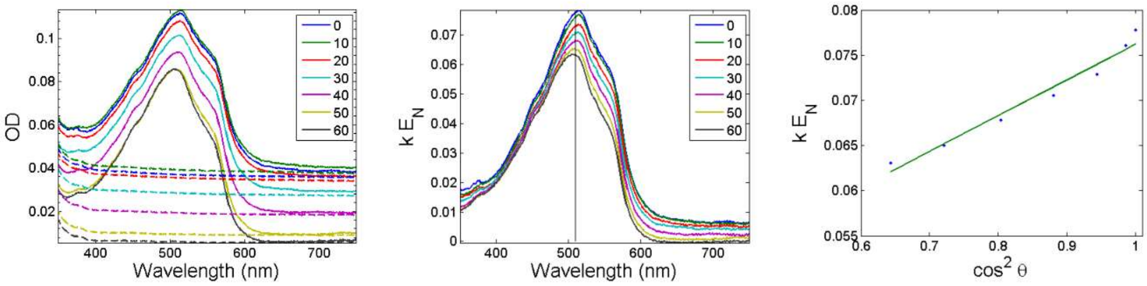

Figure S32. Left) Raw data from polarized optical spectroscopy (tilt angles from 0° to 60° in steps of 10). The spin coated films are represented with solid lines and the background from the glass with dashed lines. Center) Corrected spectra given by the constant k times the absorption coefficient E_N. Right) Linear regression at the maximum of the emission to find α_{fZ}.

Bordeaux red BZK14 Hold 8

Optical Microscopy

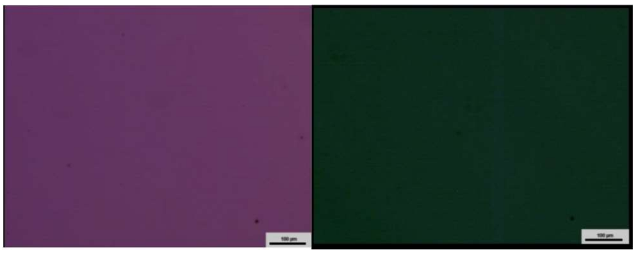

Figure S33. Wide field optical microscopy (top) and dark field microscopy (bottom) images of the edge of a thin film.

Atomic Force Microscopy

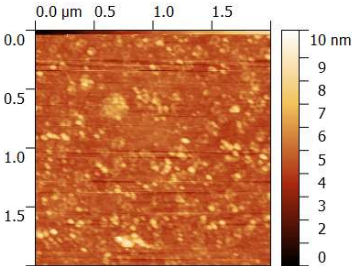

Figure S34. AFM micrograph from the central region of a thin film.

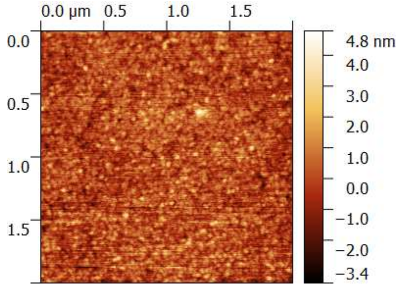

Figure S35. AFM micrograph from the central region of a thin film.

Polarized Spectroscopy

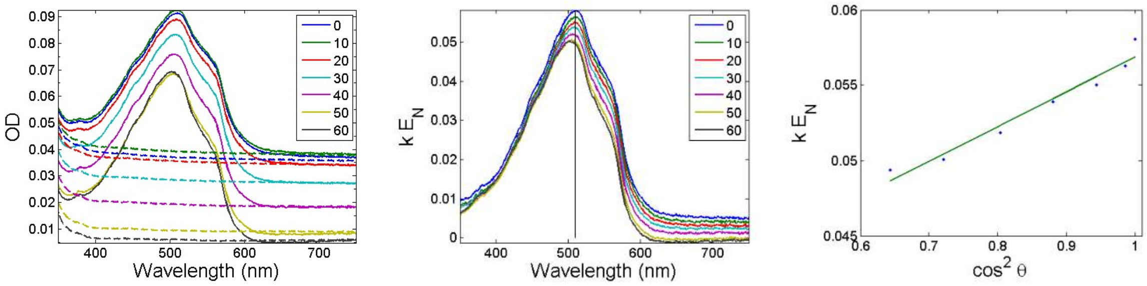

Figure S36. Left) Raw data from polarized optical spectroscopy (tilt angles from 0° to 60° in steps of 10). The spin coated films are represented with solid lines and the background from the glass with dashed lines. Center) Corrected spectra given by the constant k times the absorption coefficient E_N. Right) Linear regression at the maximum of the emission to find α_{fZ}.

Bordeaux red BZK16 Hold 11

Optical Microscopy

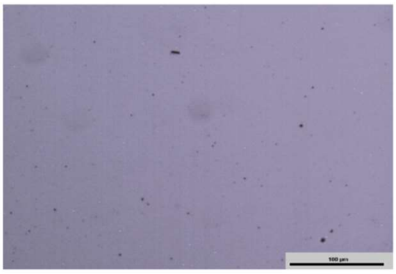

Figure S37. Wide field optical microscopy image of the central region of a thin film.

Atomic Force Microscopy

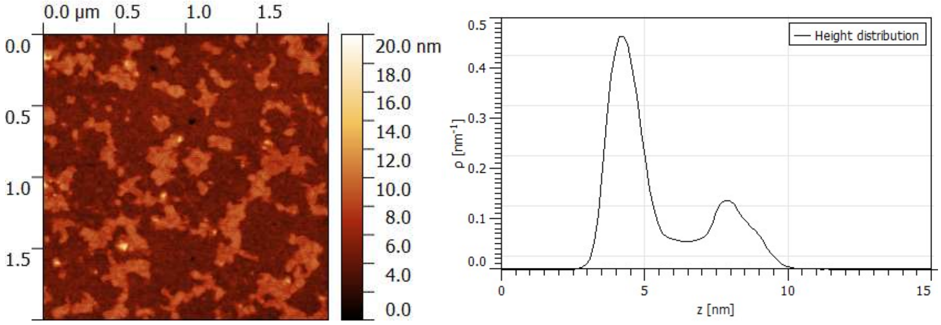

Figure S38. AFM micrograph and height distribution (entire image) from the central region of a thin film.

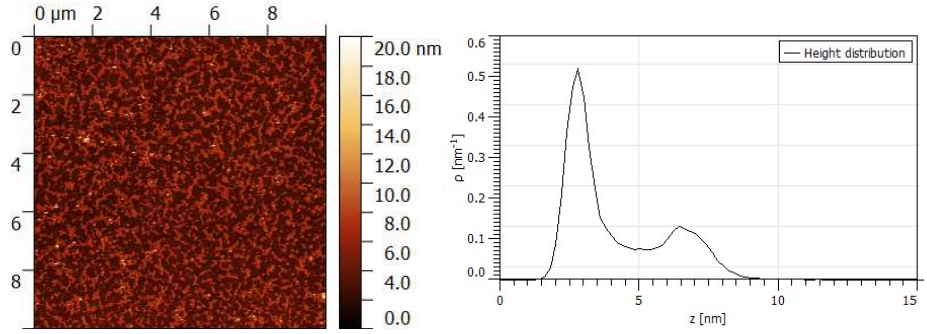

Figure S39. AFM micrograph and height distribution (entire image) from the central region of a thin film.

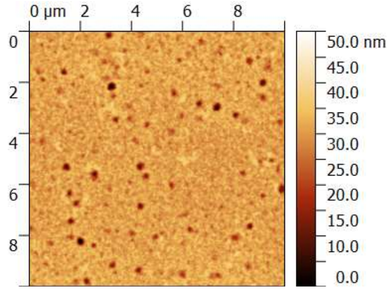

Figure S40. AFM micrograph from the central region of a thin film.

Polarized Spectroscopy

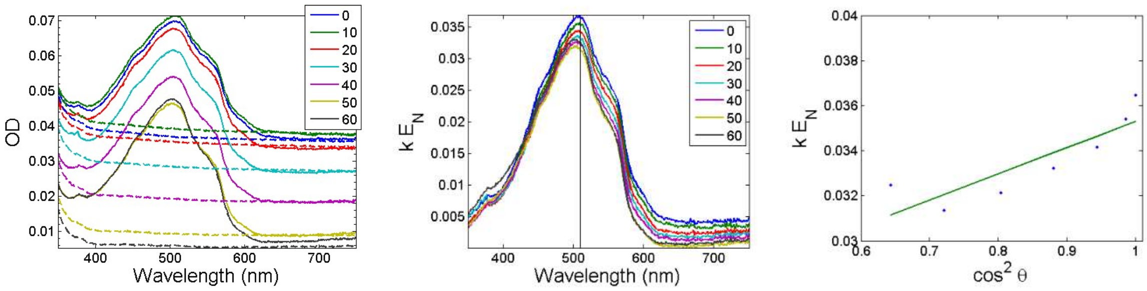

Figure S41. Left) Raw data from polarized optical spectroscopy (tilt angles from 0° to 60° in steps of 10). The spin coated films are represented with solid lines and the background from the glass with dashed lines. Center) Corrected spectra given by the constant k times the absorption coefficient E_N. Right) Linear regression at the maximum of the emission to find α_{fz}.

Bordeaux red BZK18 Hold 16

Optical Microscopy

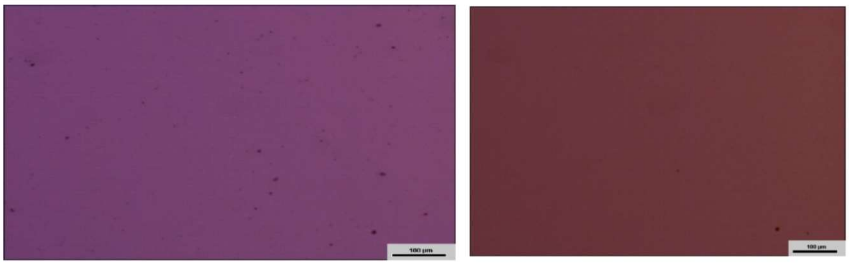

Figure S42. Wide field optical microscopy (top) and dark field microscopy (bottom) images of the edge of a thin film.

Atomic Force Microscopy

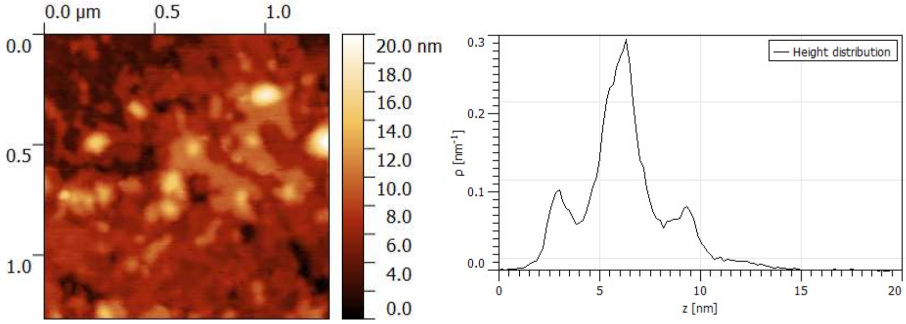

Figure S43. AFM micrograph and height distribution (entire image) from the edge of a thin film.

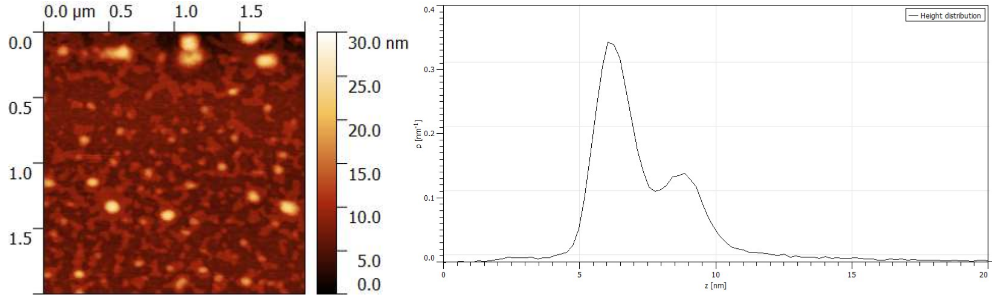

Figure S44. AFM micrograph and height distribution (entire image) from the edge of a thin film.

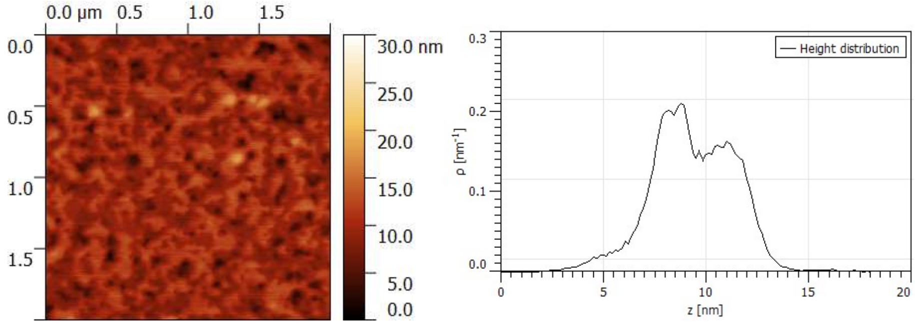

Figure S45. AFM micrograph and height distribution (entire image) from the central region of a thin film.

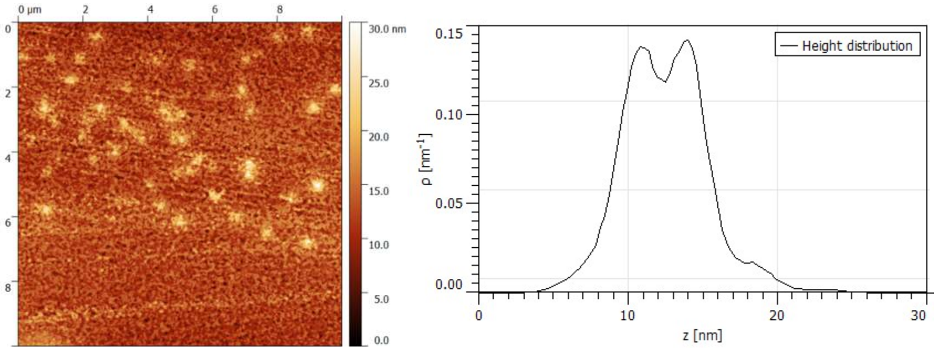

Figure S46. AFM micrograph and height distribution (entire image) from the central region of a thin film.

Polarized Spectroscopy

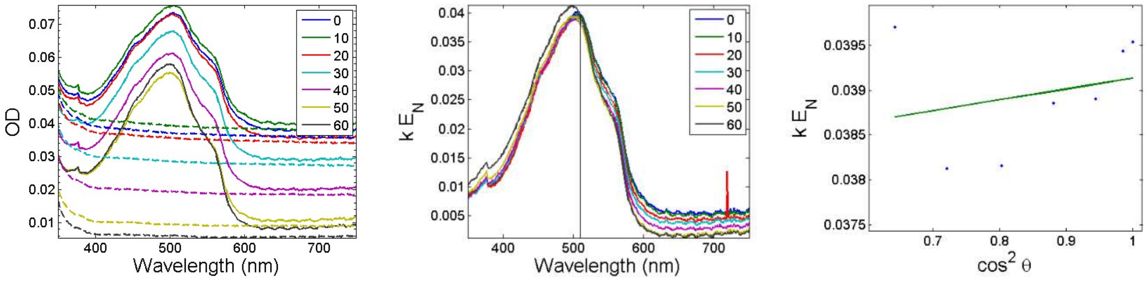

Figure S47. (**Left**) Raw data from polarized optical spectroscopy (tilt angles from 0° to 60° in steps of 10). The spin coated films are represented with solid lines and the background from the glass with dashed lines. (**Center**) Corrected spectra given by the constant k times the absorption coefficient E_N. (**Right**) Linear regression at the maximum of the emission to find α_{fZ}.

Amaranth BZK10 Hold 3

Optical Microscopy

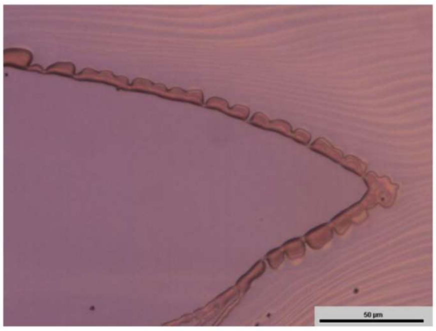

Figure S48. Wide field optical microscopy image of the edge of a thin film.

Atomic Force Microscopy

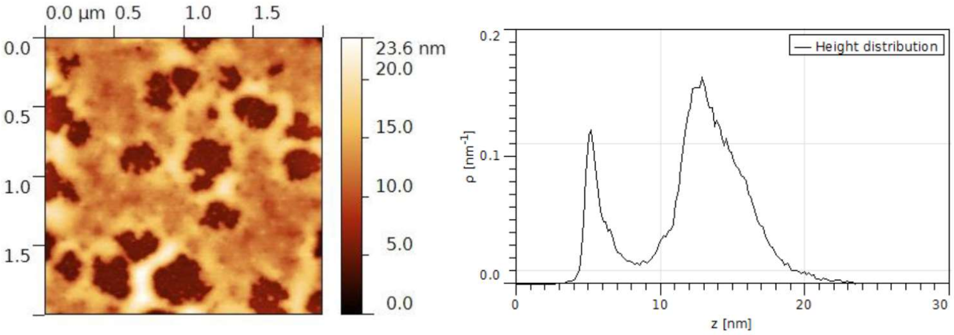

Figure S49. AFM micrograph and height distribution (entire image) from the central region of a thin film.

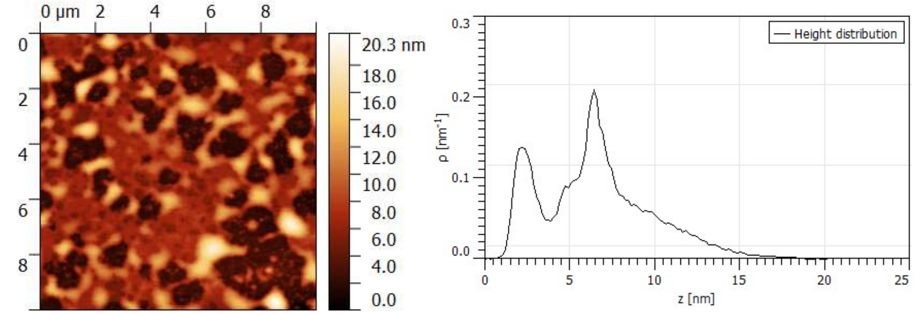

Figure S50. AFM micrograph and height distribution (entire image) from the central region of a thin film.

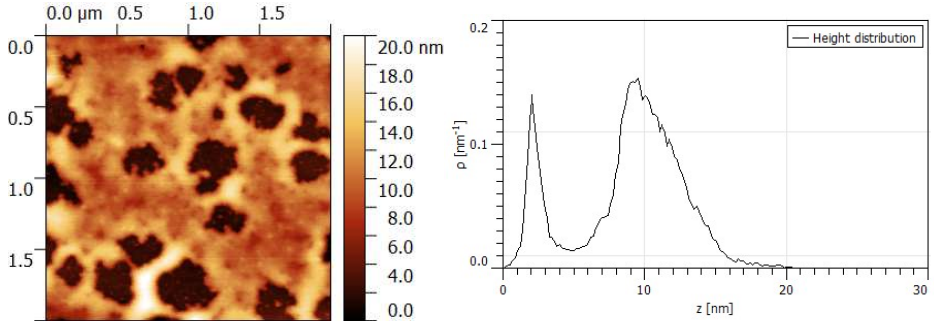

Figure S51 AFM micrograph and height distribution (entire image) from the central region of a thin film.

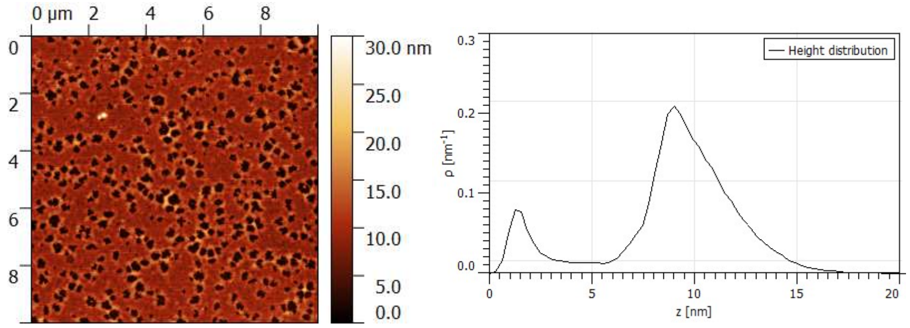

Figure S52. AFM micrograph and height distribution (entire image) from the central region of a thin film.

Polarized Spectroscopy

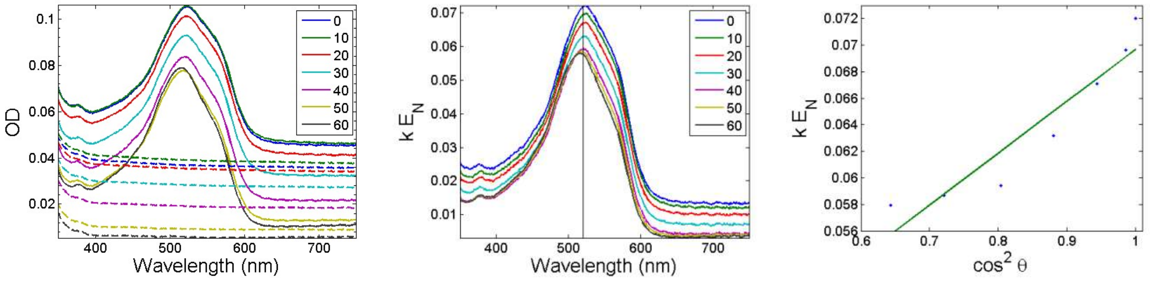

Figure S53. Left) Raw data from polarized optical spectroscopy (tilt angles from 0° to 60° in steps of 10). The spin coated films are represented with solid lines and the background from the glass with dashed lines. Center) Corrected spectra given by the constant k times the absorption coefficient E_N. Right) Linear regression at the maximum of the emission to find α_{fZ}.

Amaranth BZK12 Hold 1

Optical Microscopy

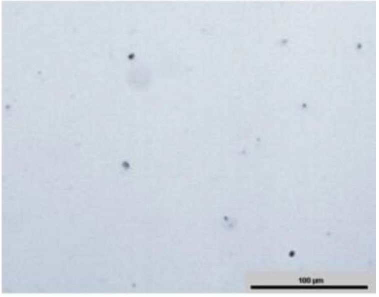

Figure S54. Wide field optical microscopy image of the central region of a thin film.

Atomic Force Microscopy

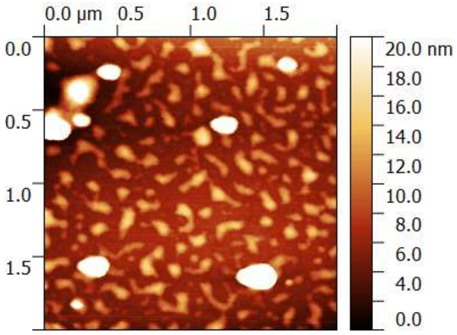

Figure S55. AFM micrograph from the central region of a thin film.

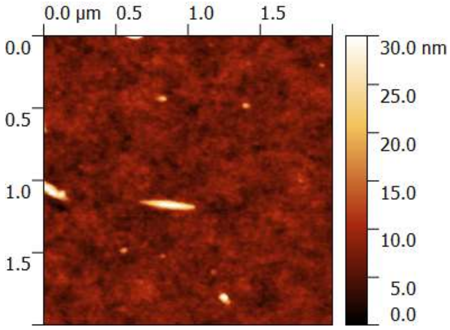

Figure S56. AFM micrograph from the central region of a thin film.

Polarized Spectroscopy

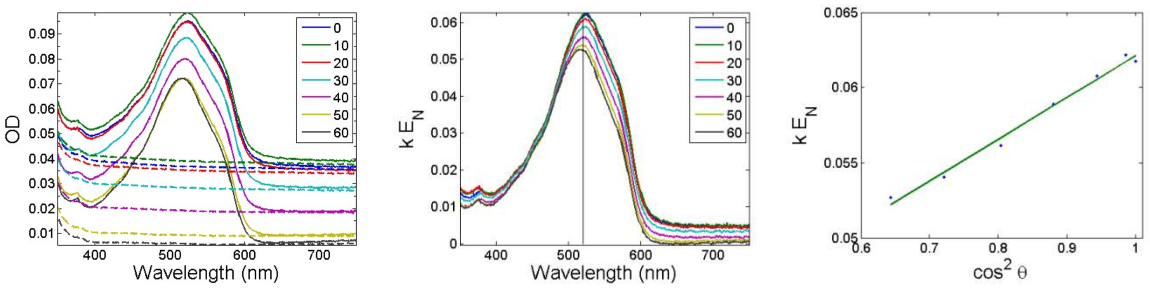

Figure S57. Left) Raw data from polarized optical spectroscopy (tilt angles from 0° to 60° in steps of 10). The spin coated films are represented with solid lines and the background from the glass with

dashed lines. Center) Corrected spectra given by the constant k times the absorption coefficient E_N. Right) Linear regression at the maximum of the emission to find α_{fZ}.

Amaranth BZK14 Hold 6

Optical Microscopy

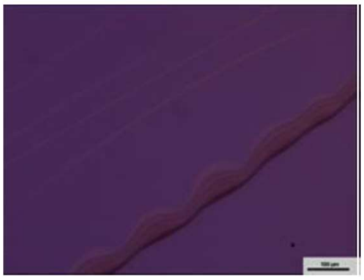

Figure S58. Wide field optical microscopy image of the edge of a thin film.

Atomic Force Microscopy

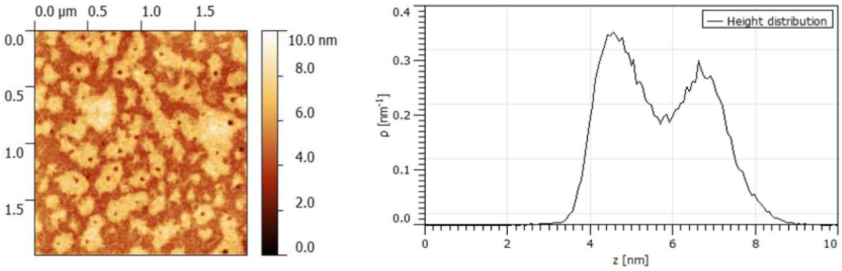

Figure S59. AFM micrograph and height distribution (entire image) from the central region of a thin film.

Polarized Spectroscopy

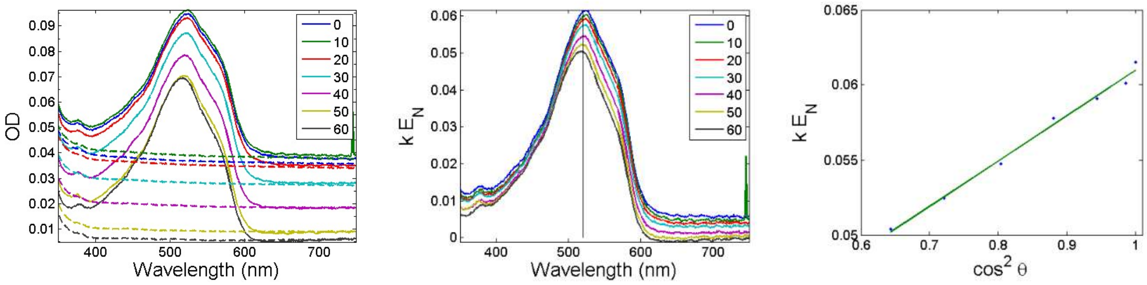

Figure S60. Left) Raw data from polarized optical spectroscopy (tilt angles from 0° to 60° in steps of 10). The spin coated films are represented with solid lines and the background from the glass with dashed lines. Center) Corrected spectra given by the constant k times the absorption coefficient E_N. Right) Linear regression at the maximum of the emission to find α_{fZ}.

Amaranth BZK16 Hold 5

Optical Microscopy

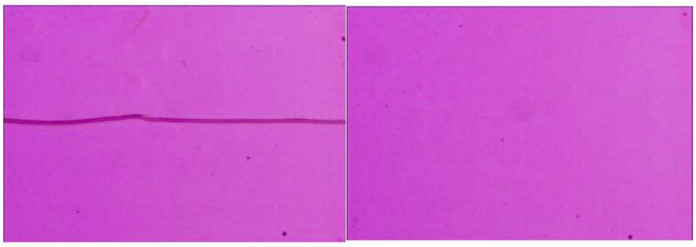

Figure S61. Wide field optical microscopy images of the edge (top) and central region (bottom) of a thin film.

Atomic Force Microscopy

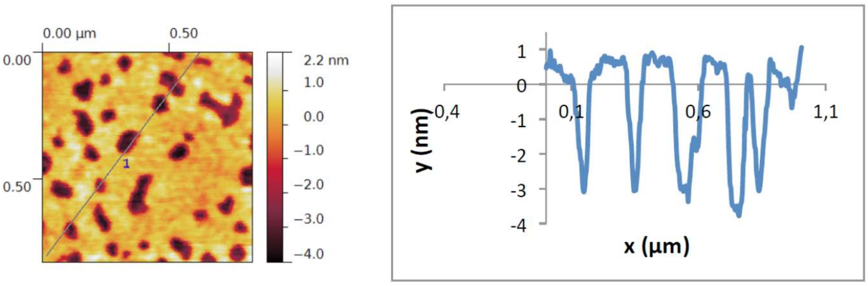

Figure S62. AFM micrograph and height profile (see line in image) from the central region of a thin film.

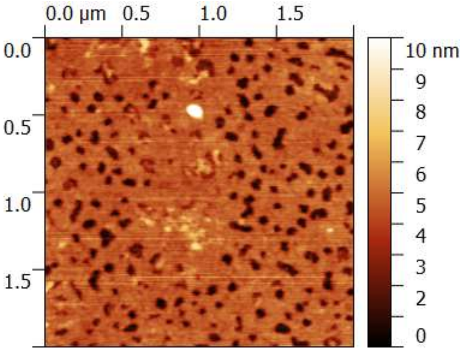

Figure S63. AFM micrograph from the central region of a thin film.

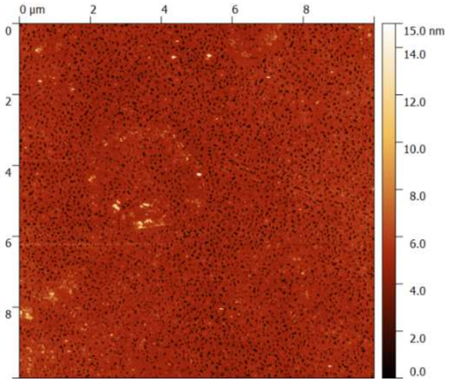

Figure S64. AFM micrograph from the central region of a thin film.

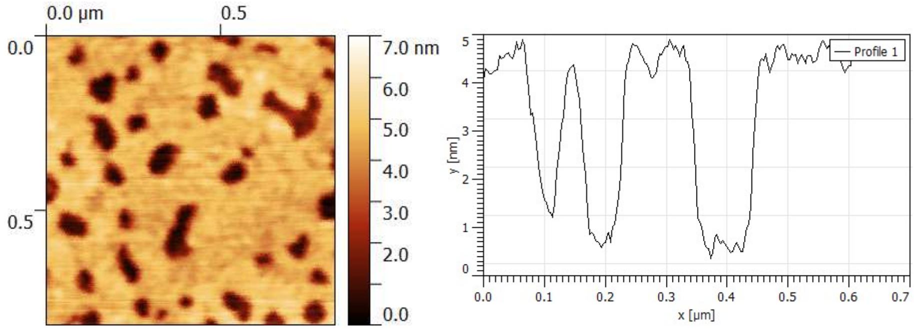

Figure S65. AFM micrograph and height profile from the central region of a thin film.

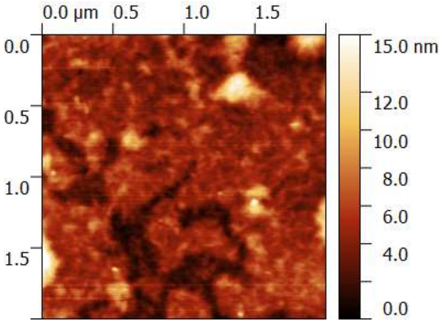

Figure S66. AFM micrograph from the edge of a thin film. Polarized Spectroscopy.

Polarized Spectroscopy

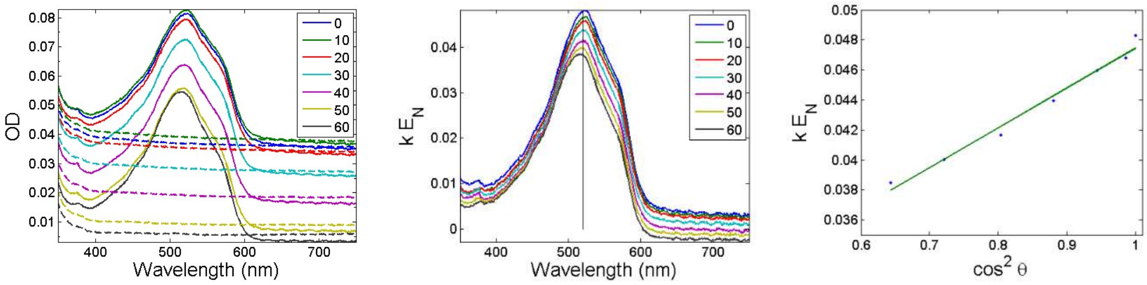

Figure S67. Left) Raw data from polarized optical spectroscopy (tilt angles from 0° to 60° in steps of 10). The spin coated films are represented with solid lines and the background from the glass with dashed lines. Center) Corrected spectra given by the constant k times the absorption coefficient E_N. Right) Linear regression at the maximum of the emission to find α_{fZ}.

Amaranth BZK18 Hold 2

Optical Microscopy

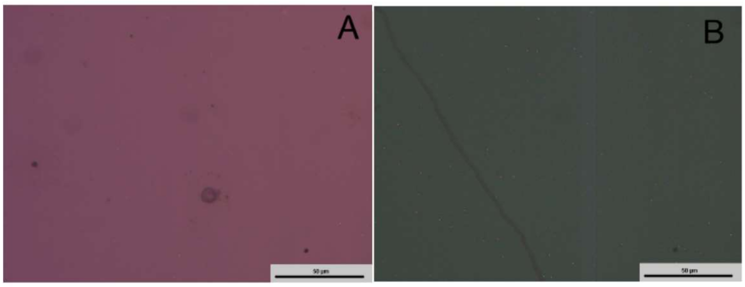

Figure S68. Wide field optical microscopy images of the edge (top) and central region (bottom) of a thin film.

Atomic Force Microscopy

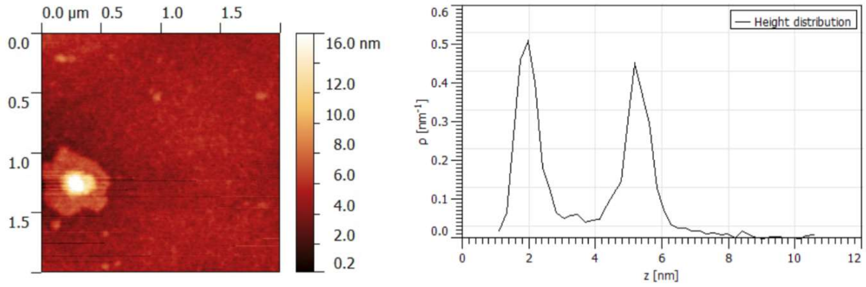

Figure S69. AFM micrograph and height distribution (part of image) from the central region of a thin film.

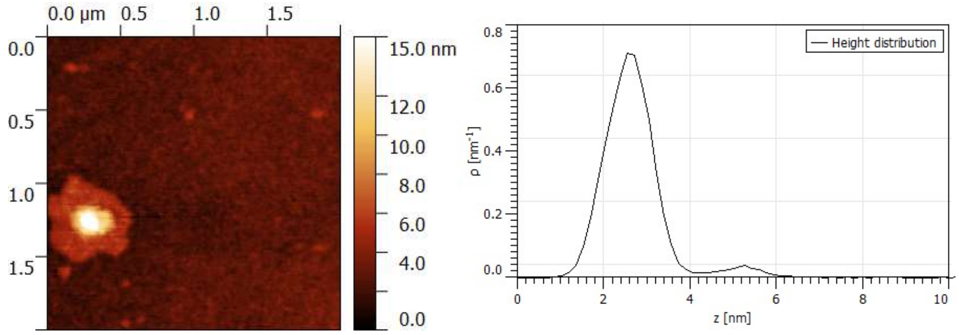

Figure S70. AFM micrograph and height distribution (entire image) from the central region of a thin film.

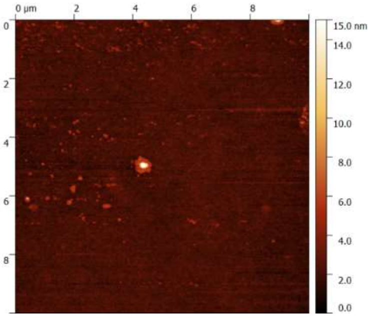

Figure S71. AFM micrograph from the central region of a thin film.

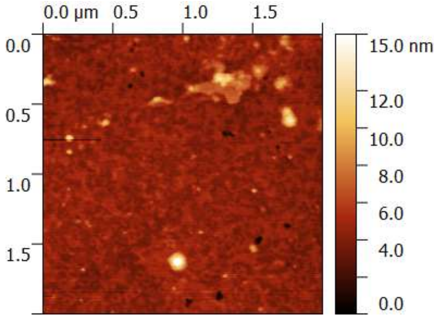

Figure S72. AFM micrograph from the central region of a thin film.

Polarized Spectroscopy

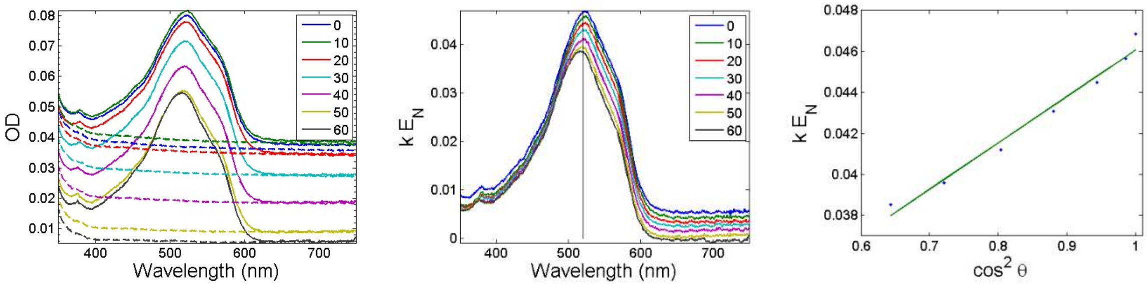

Figure S73. Left) Raw data from polarized optical spectroscopy (tilt angles from 0° to 60° in steps of 10). The spin coated films are represented with solid lines and the background from the glass with dashed lines. Center) Corrected spectra given by the constant k times the absorption coefficient E_N. Right) Linear regression at the maximum of the emission to find α_{fZ}.

Trypan Blue BZK10 Hold 7

Optical Microscopy

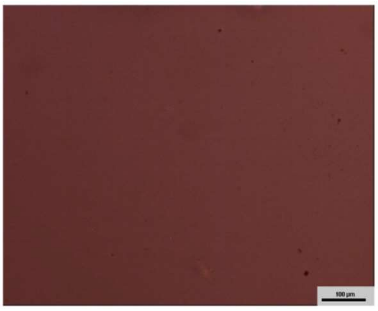

Figure S74. Wide field optical microscopy image of the central region of a thin film.

Atomic Force Microscopy

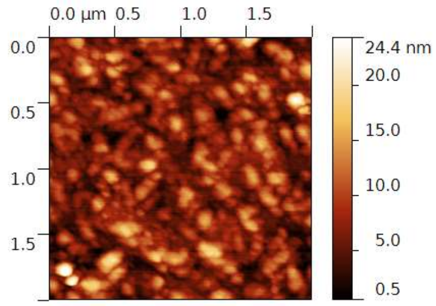

Figure S75. AFM micrograph from the central region of a thin film.

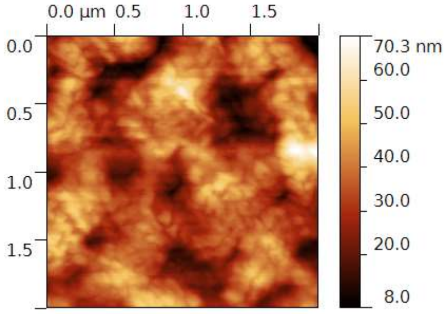

Figure S76. AFM micrograph from the central region of a thin film.

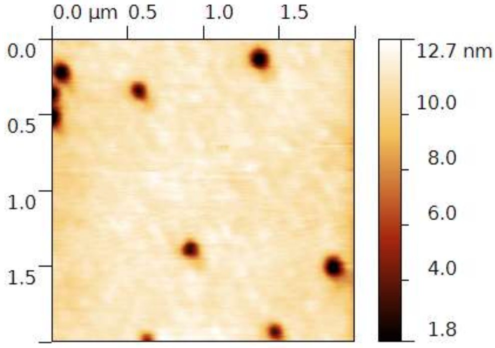

Figure S77. AFM micrograph from the central region of a thin film.

Polarized Spectroscopy

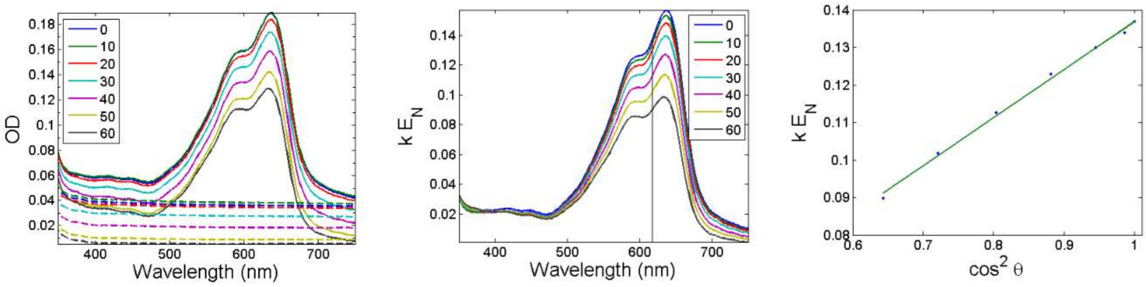

Figure S78. Left) Raw data from polarized optical spectroscopy (tilt angles from 0° to 60° in steps of 10). The spin coated films are represented with solid lines and the background from the glass with dashed lines. Center) Corrected spectra given by the constant k times the absorption coefficient E_N. Right) Linear regression at the maximum of the emission to find α_{fZ}.

Polarised Spectroscopy Analysis

Figure 4 in the main text shows the data and the result from the modelling of the experimental data for Allura Red in stretched polymers. Here, the α_{fz} angle is well defined at 48°, while the ϕ_{fz} angle can vary from ≈30° to ≈60° at the emission maximum, depending on the assumptions made regarding the overall shape of the dye molecule. The assumptions regarding the molecular shape correspond to assuming that the molecules at perfect alignment can be located at a specific point of the orientation triangle. The relation between ϕ_{fz}, the assumed shape of the molecule, and the orientation factor is plotted in Figure 4 in the main text and in Figure S79 below, using equation 5. If the molecule is considered to be rod-like it will follow the top line of the orientation triangle, while a flat-like molecule will be at the bottom edge of the orientation triangle. The numerical analysis allows us to determine the possible values ϕ_{fz} can take as a function of the position in the orientation triangle.

To analyse the data an assumption of the degree of alignment must be made. With intermediate limiting alignment (region R2) the angle between the transition moment and the stretch direction must be within the range 28°≤ϕ_{fz}≤68°. If the limiting alignment with the stretch direction is better (R1) we can predict that the angle must be in the region 43°≤ϕ_{fz}≤53, and if the molecule can be perfectly aligned we can determine that ϕ_{fz} will be 48°. These considerations are compiled in Table S1 for all four dyes, while figure S79 shows the orientation triangles and corresponding ϕ_{fz} vales for Allura Red and Trypan Blue. Note that the gray areas in figure S79 are excluded due to the experimentally determined values of k_f.

For the purpose of describing the direction of the transition moment in the molecular structure the data is not perfect. For the box-like molecules Allura Red, Bordeaux Red, and Amaranth we can estimate a ≈ 10° range where ϕ_{fz} must lie within when we assume an alignment of 0.7≤kz≤1 (region R1, Figure S79). For the rod-shaped Trypan Blue a higher spread of 33° is found for that range of kz. A much narrower 4 degree range is found if we assume a higher degree of alignment 0.9≤kz≤1 (region R1, Figure S79) than for the other molecules, due to the well-defined shape of the molecule. For the purpose of determining the molecular structure of thin films these results clearly show the orientation of the transition moments ($\mathbf{M}_f$) with respect to the molecular long axis (z). The detailed data is compiled in Table S1.

Trypan Blue provides a clear example. In stretched PVA the rod-like molecule is oriented with z and Z close to the same axis i.e. k_z is close to 1. In the thin films, the Z axis is perpendicular to the lamellar, such that Trypan Blue is oriented with the long axis (z) roughly perpendicular to Z. The data shows that $\alpha_{fZ,\mathrm{PVA}}$ = 33° while $\alpha_{fZ,\mathrm{thin\ film}}$ = 80° , which assuming ideal alignment $\omega_{zZ,\mathrm{PVA}}$ ≈ 0° $\phi_{fz,\mathrm{PVA}}$ ≈ 33° corresponds to $\omega_{zZ,\mathrm{thin\ film}}$ at either ≈53° or ≈113°. From a fundamental standpoint a large different, but as can be seen in Figure 4, the molecular structure in the two materials that would give rise to the different angles is small. As for Trypan Blue $\phi_{fz,\mathrm{PVA}}$ =33° is the maximum $\phi_{fz,\mathrm{PVA}}$ angle, when considering $\omega_{zZ,\mathrm{PVA}}$ ≠ 0° we can provide the boundary 53°≤$\omega_{zZ,\mathrm{thin}}$≤113°.

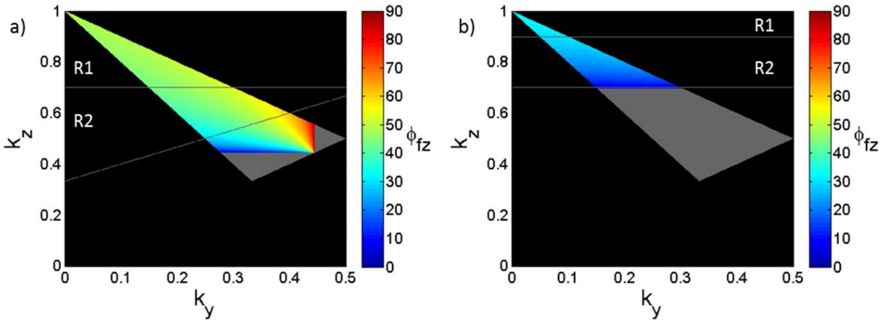

Figure S79. Orientation triangles for Allura Red (left) and Trypan Blue (right) showing the relation between ϕ_{fz} and the orientation factors is. ϕ_{fz} angle (color) satisfies the requirement $k_z \geq k_f \geq k_y$ from

Equation 5. The area of orientation triangle shaded gray is excluded by the experimentally determined value of k_f.

Table S1. Summary of the results from the numerical analysis of polarized spectroscopy data obtained on azo-dyes in stretched PVA. The results are presented as a function of the orientation factor (k_z) and the assumed shape of the molecular structure. The insert show the orientation of the transition moment (M_f) in the molecular structure of Allura Red, Bordeaux Red, Amaranth, and Trypan Blue in the case of perfect alignment ($\alpha_{fZ} = \phi_{fz}$) represented with black arrows. And in case of intermediate alignment assuming that the molecular shape is rod-like (blue arrows) or flat (red arrows).

	**Allowed orientation factor range**	**Alura Red ϕ_{fz},max**	**Bordeux Red ϕ_{fz},max**	**Amarant ϕ_{fz},max**	**Trypan Blue ϕ_{fz},max**
λ		514 nm	530 nm	534 nm	617 nm
Experimental k_f[a]		0.4441	0.4291	0.4593	0.6966
Perfect alignment $\boldsymbol{\alpha_{fZ}} = \boldsymbol{\phi_{fz}}$	$k_z = 1$	48	49	47	33
Assuming intermediate alignment	$k_z > 0.7$	43–53	45–55	41–51	29–33[b]
Possible orientation angles	$0.5/0.6 > k_z > 1$[c]	28–62	32–68	24–57	4–33[b]

[a] The number of decimals indicated does not represent the accuracy of calculating k_f , however it is important to take all the decimals in order to calculate the angles consistently. Note that the angles are not presented with such a high accuracy. [b] The experimental k_z is 0.6966 for trypan blue, the interval reported are for $k_z > 0.9$ and $k_z > 0.7$. [c] The region excluded by the experimental values of k_f is asymmetric in kz. The interval is selected from kz = 0.5 in the rod-like limit and $k_z = 0.6$ in the flat-like limit.

Stretched polymer analysis

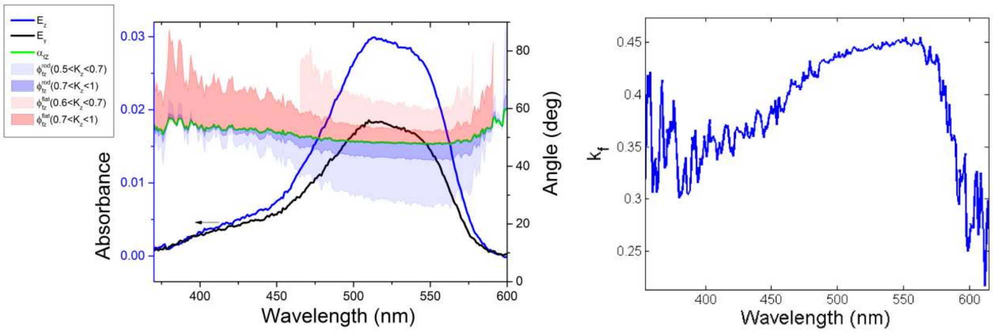

Figure S80. Left: Angles respect the stretching direction of Alura Red in polyvinyl alcohol. Right: Orientation factor k_f of the transition $\boldsymbol{M}_f$ respect to the stretching direction Z.

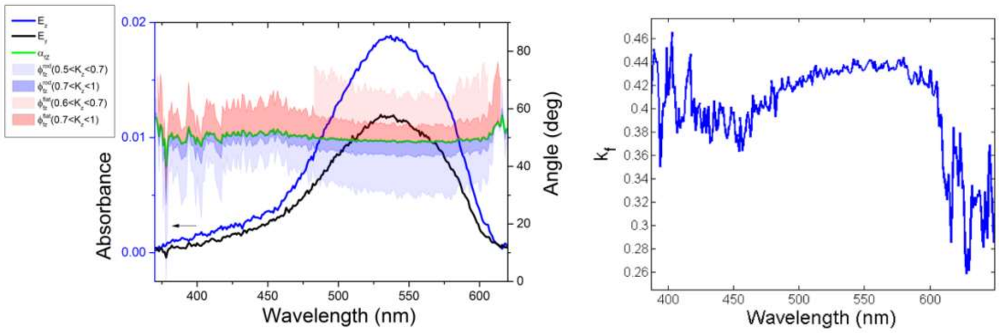

Figure S81. Left: Angles respect the stretching direction of Bordeaux Red in polyvinyl alcohol. Right: Orientation factor k_f of the transition $\boldsymbol{M}_f$ respect to the stretching direction Z.

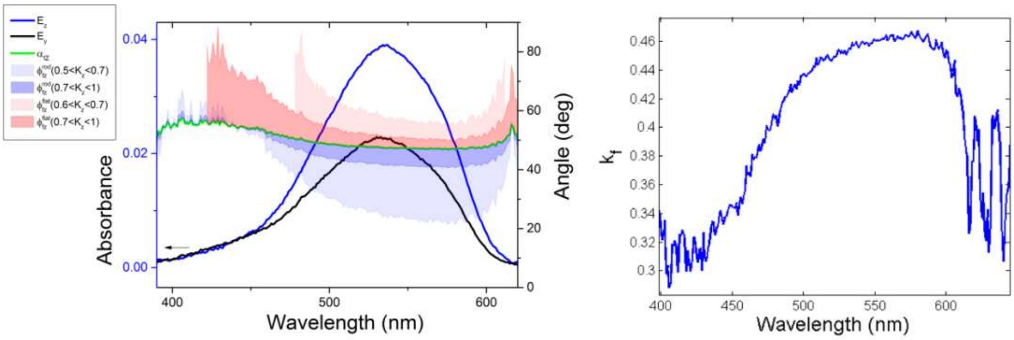

Figure S82. Left: Angles respect the stretching direction of Amarant in polyvinyl alcohol. Right: Orientation factor k_f of the transition $\boldsymbol{M}_f$ respect to the stretching direction Z.

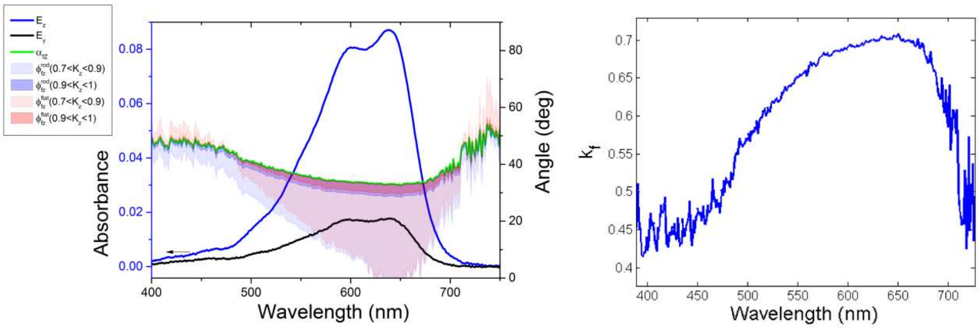

Figure S83. Left: Angles respect the stretching direction of Trypan Blue in polyvinyl alcohol. Right: Orientation factor k_f of the transition $\boldsymbol{M}_f$ respect to the stretching direction Z.

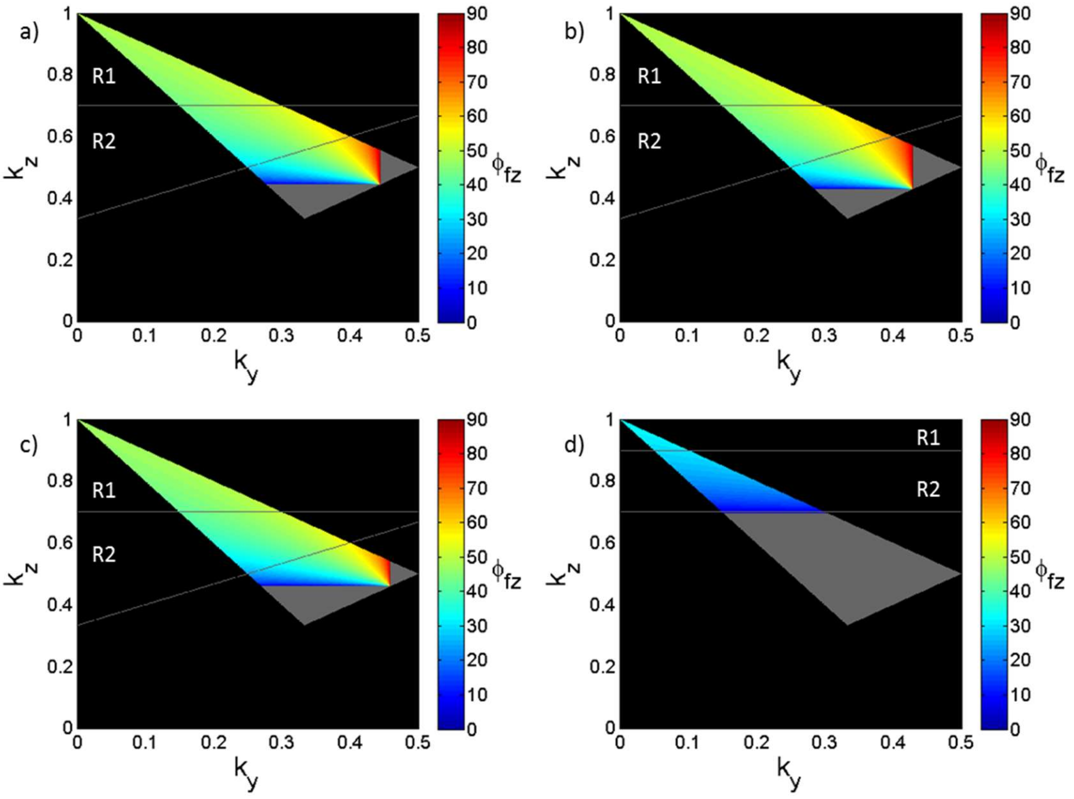

Figure S84. (**a**) Allura Red; (**b**) Bordeaux Red; (**c**) Amarant; (**d**) Tryphan Blue.

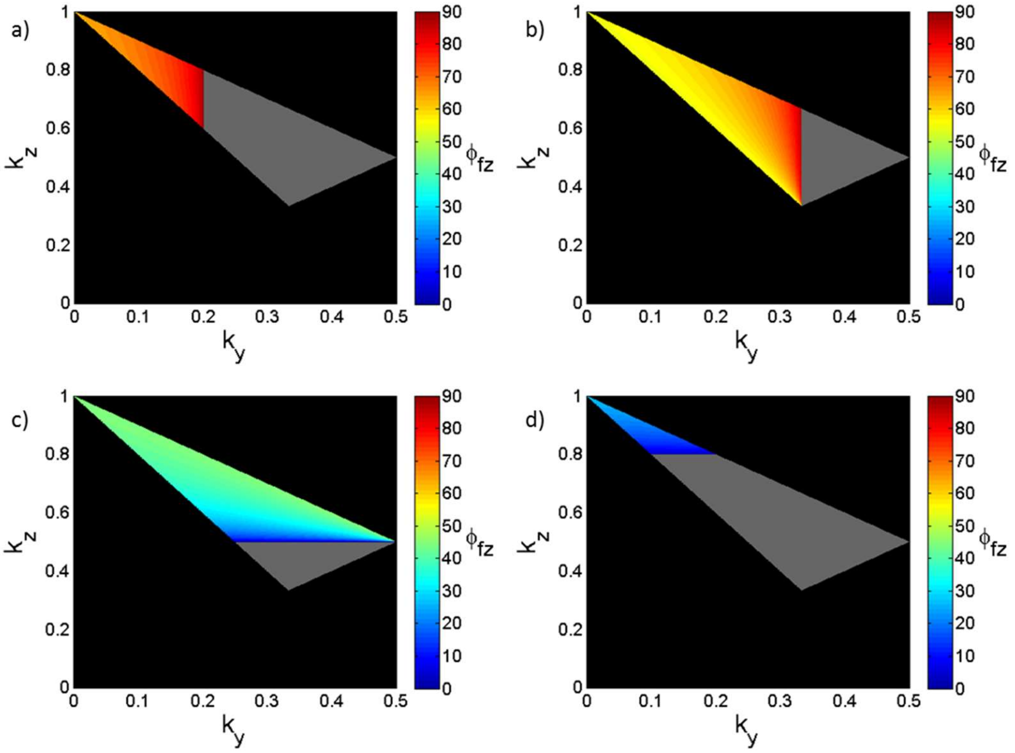

Figure S85. a) k_f=0.2b) k_f=1/3 c) k_f=0.5 d) k_f=0.8.

nanomaterials

Table S2. Angle between transition dipole and long molecule axes assuming planar molecules and/or rod-like orientation.

k_f	ϕ_{fz}min ≤ϕ_{fz}≤ϕ_{fz}max (all posible values in the orientation triangle)
0.2	63.4°≤ϕ_{fz}≤90°
0.3333	54.7°≤ϕ_{fz}≤90°
0.5	0°≤ϕ_{fz}≤45°
0.8	0°≤ϕ_{fz}≤26.6°
0.4441 (Allura Red)	0°≤ϕ_{fz}≤90°
0.4291 (Bordeaux Red)	0°≤ϕ_{fz}≤90°
0.4593 (Amarant)	0°≤ϕ_{fz}≤90°
0.6966 (Tryphan Blue)	0°≤ϕ_{fz}≤33°

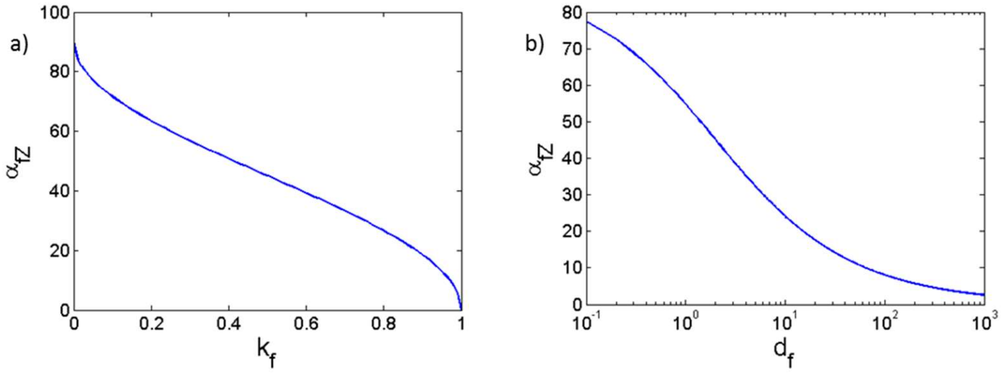

Figure S86. (**a**) Transition dipole angle respect to the uniaxial axes Z against k_f; (**b**) Transition dipole angle respect to the uniaxial axes Z against the dichroic ratio d_f.

© 2018 by the authors. Submitted for possible open access publication under the terms and conditions of the Creative Commons Attribution (CC BY) license (http://creativecommons.org/licenses/by/4.0/).
